# Carbon Dioxide Concentration Mechanisms in Natural Populations of Marine Diatoms: Insights From *Tara* Oceans

**DOI:** 10.3389/fpls.2021.657821

**Published:** 2021-04-30

**Authors:** Juan José Pierella Karlusich, Chris Bowler, Haimanti Biswas

**Affiliations:** ^1^Institut de Biologie de l’ENS, Département de Biologie, École Normale Supérieure, CNRS, INSERM, Université PSL, Paris, France; ^2^CNRS Research Federation for the study of Global Ocean Systems Ecology and Evolution, FR2022/Tara Oceans GOSEE, Paris, France; ^3^CSIR National Institute of Oceanography, Biological Oceanography Division, Dona Paula, India

**Keywords:** *Tara* Oceans, diatoms, carbon metabolism, carbon dioxide concentration mechanisms, metagenomics, metatranscriptomics

## Abstract

Marine diatoms, the most successful photoautotrophs in the ocean, efficiently sequester a significant part of atmospheric CO_2_ to the ocean interior through their participation in the biological carbon pump. However, it is poorly understood how marine diatoms fix such a considerable amount of CO_2_, which is vital information toward modeling their response to future CO_2_ levels. The *Tara* Oceans expeditions generated molecular data coupled with *in situ* biogeochemical measurements across the main ocean regions, and thus provides a framework to compare diatom genetic and transcriptional flexibility under natural CO_2_ variability. The current study investigates the interlink between the environmental variability of CO_2_ and other physicochemical parameters with the gene and transcript copy numbers of five key enzymes of diatom CO_2_ concentration mechanisms (CCMs): Rubisco activase and carbonic anhydrase (CA) as part of the physical pathway, together with phosphoenolpyruvate carboxylase, phosphoenolpyruvate carboxykinase, and malic enzyme as part of the potential C4 biochemical pathway. Toward this aim, we mined >200 metagenomes and >220 metatranscriptomes generated from samples of the surface layer of 66 globally distributed sampling sites and corresponding to the four main size fractions in which diatoms can be found: 0.8–5 μm, 5–20 μm, 20–180 μm, and 180–2,000 μm. Our analyses revealed that the transcripts for the enzymes of the putative C4 biochemical CCM did not in general display co-occurring profiles. The transcripts for CAs were the most abundant, with an order of magnitude higher values than the other enzymes, thus implying the importance of physical CCMs in diatom natural communities. Among the different classes of this enzyme, the most prevalent was the recently characterized iota class. Consequently, very little information is available from natural diatom assemblages about the distribution of this class. Biogeographic distributions for all the enzymes show different abundance hotspots according to the size fraction, pointing to the influence of cell size and aggregation in CCMs. Environmental correlations showed a complex pattern of responses to CO_2_ levels, total phytoplankton biomass, temperature, and nutrient concentrations. In conclusion, we propose that biophysical CCMs are prevalent in natural diatom communities.

## Introduction

**FIGURE 1:**
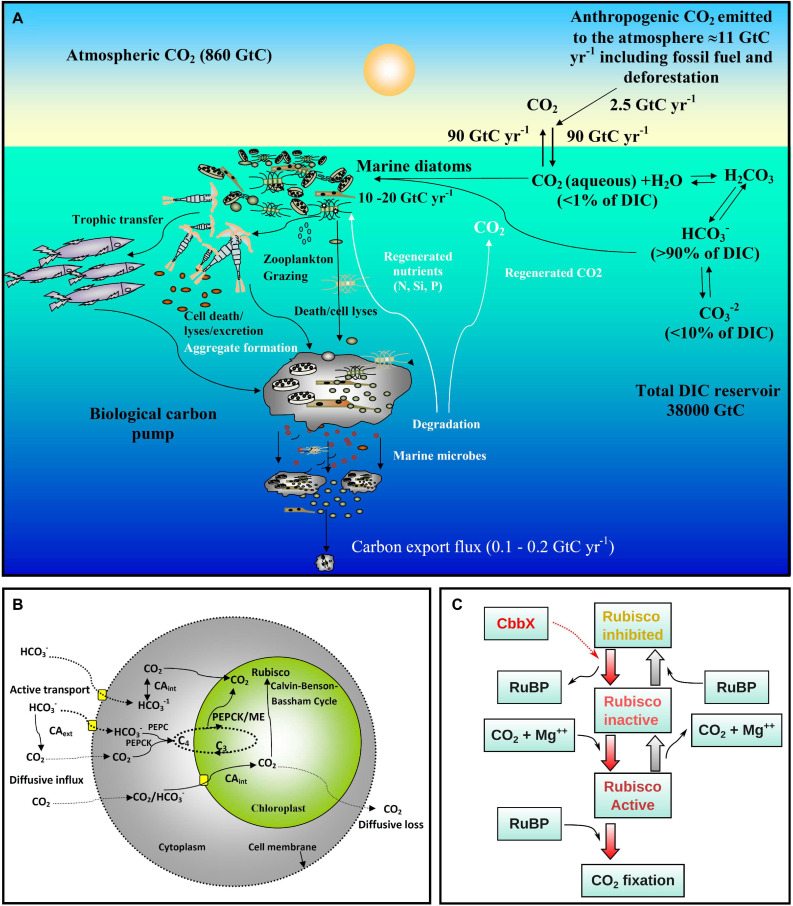
Overview of ocean carbon cycle and diatom carbon dioxide concentration mechanisms. **(A)** Schematic representation of the ocean carbon cycle depicting the role of marine diatoms in the biological carbon pump. The anthropogenic CO_2_ emission to the atmosphere (mainly generated by fossil fuel burning and deforestation) is nearly 11 Gigaton carbon (GtC) per year, of which almost 2.5 GtC is taken up by the surface ocean. In surface seawater (pH 8.1–8.4), bicarbonate (HCO_3_^–^) and carbonate ions (CO_3_^2–^) constitute nearly 90 and <10% of dissolved inorganic carbon (DIC) respectively, while dissolved CO_2_ (CO_2_ aqueous) contributes <1%. Despite this low level of CO_2_ in the ocean and its slow diffusion rate in water, diatoms fix 10–20 GtC annually via photosynthesis thanks to their carbon dioxide concentration mechanisms (CCMs), allowing them to sustain food chains. In addition, 0.1–1% of this organic material produced in the euphotic layer sinks down as particles, thus transferring the surface carbon toward the deep ocean and sequestering atmospheric CO_2_ for thousands of years or longer. The remaining organic matter is remineralized through respiration. Thus, diatoms are one of the main players in this biological carbon pump, which is arguably the most important biological mechanism in the Earth System allowing CO_2_ to be removed from the carbon cycle for very long period. Based on data from Friedlingstein et al., 2020. **(B)** Schematic representation of the CCMs in diatoms. The low levels of CO_2_ in the ocean and its slow diffusion rate in water have led diatoms and other photosynthetic organisms to evolve CCMs that utilize the higher concentrations of HCO_3_^–^. The biophysical CCM consists of various bicarbonate transporters and carbonic anhydrases (CAs) that serve to increase the CO_2_ flux balance toward the pyrenoid, a low CO_2_-permeable subcellular compartment in the chloroplast containing most of the Rubisco. In addition, some diatoms may also have a biochemical (C4-like) CCM involving phosphoenolpyruvate carboxylase (PEPC), phosphoenolpyruvate carboxykinase (PEPCK) and/or malic enzyme (ME). **(C)** Schematic presentation of Rubisco activation by CbbX in diatoms and other phototrophs with red-type Rubisco. CbbX functions as a mechanochemical motor protein and uses the energy from ATP hydrolysis to modify the structure of Rubisco. This process facilitates the dissociation of inhibitory sugar phosphates [ribulose-1,5-bisphosphate (RuBP) and others] from the active site of Rubisco.

Diatoms are among the most successful and diversified eukaryotic photoautotrophs in the present day ocean (Armbrust, 2009; Malviya et al., 2016; Pierella Karlusich et al., 2020). Their fast growth rates in high-nutrient environments and comparatively large sizes make them important contributors to organic carbon production. On an annual scale, marine diatoms fix 10–20 billion metric tons of inorganic carbon (comparable to all global rainforests combined), corresponding to up to 40% of the total marine primary production and as much as 20% of the total primary production on Earth (Field et al., 1998; Smetacek, 1999; Granum et al., 2005; Jin et al., 2006; Falkowski and Raven, 2013; Tréguer et al., 2018). Thus, diatoms are main contributors to marine food chains and in sequestering atmospheric CO_2_ to the ocean interior through gravitational sinking of particles (biological carbon pump) (Figure 1A), and hence have high biogeochemical significance (Tréguer et al., 2018; Boyd et al., 2019). Diatoms possess a peculiar gene complement derived from green and red algal sources, and have many genes in common with animals and bacteria (Bowler et al., 2008; Moustafa et al., 2009; Dorrell et al., 2017, 2021), mostly owing to their chimeric evolutionary origins as well as to horizontal gene transfer events (Armbrust, 2009; Dorrell et al., 2021). It is believed that these genes have enabled them to develop unique and highly efficient carbon (Schoefs et al., 2017) and nitrogen metabolism pathways (Wilhelm et al., 2006; Busseni et al., 2019). In the present context of climate change and the substantial anthropogenic perturbations in the ocean (increasing CO_2_ and temperature, acidification, disturbances in nutrient cycles, etc.), a key question is how marine diatoms will respond. In order to do this, a clear understanding of diatom carbon metabolism is required.

The key carbon fixing enzyme, ribulose-1,5-bisphosphate carboxylase/oxygenase, Rubisco, is one of the most abundant proteins on Earth and is responsible for 100 billion tons of carbon fixation annually (Erb and Zarzycki, 2018; Bar-On and Milo, 2019). Remarkably however, Rubisco is highly inefficient because it shows specificity toward O_2_ which competes with CO_2_. This is believed to be a remnant of its evolution at a time when oxygen levels were minimal, and leads to a wasteful process called photorespiration (Poudel et al., 2020). Diatoms possess a red algal type Rubisco (type ID) which is one of the most efficient Rubisco forms, with the highest preference toward carboxylation over oxygenation (Young et al., 2016). However, CO_2_ concentration in the present day surface oceans is on average 10–12 μmol kg^–1^ [<1% of the dissolved inorganic carbon (DIC) pool] (Figure 1A), which is well below the half saturation constant of Rubisco for CO_2_ (Badger et al., 1998). Instead, the DIC pool is >90% bicarbonate ions (HCO_3_^–^) (Figure 1A). To optimize carboxylation even at present CO_2_ levels, most photoautotrophs have developed active carbon dioxide concentration mechanisms (CCMs) which can be biophysical or biochemical (Figure 1B). Such CCMs aim to maintain a higher CO_2_ concentration over O_2_ in the vicinity of Rubisco (Reinfelder, 2011). In a biophysical CCM, the cells actively pump bicarbonate ions (HCO_3_^–^) inside the cell followed by the conversion of HCO_3_^–^ to CO_2_ (Hopkinson et al., 2011; Reinfelder, 2011) via the metalloenzyme carbonic anhydrase (CA; Morel et al., 1994; Badger, 2003). CO_2_ molecules enter the cell through the lipid bilayer membrane (Gutknecht et al., 1977) and can easily diffuse out due to the high concentration gradient. It has been proposed that in diatoms cytoplasmic CA continuously maintains low CO_2_ levels by converting CO_2_ to HCO_3_^–^ for facilitating a CO_2_ diffusive influx (Matsuda et al., 2017). Hence, CA plays a key role in carbon acquisition in diatoms. The functioning of biophysical CCMs in marine diatoms has been well studied in laboratory conditions (Hopkinson et al., 2016; reviewed by Matsuda et al., 2017) and was shown to be highly diverse and more efficient than in C4 plants (Young et al., 2016). Down regulation of CCM/photorespiratory genes under elevated CO_2_ levels in model marine diatoms has been observed in experimental studies (Ohno et al., 2012; Hennon et al., 2015; Li et al., 2015).

In biochemical CCMs, the enzyme phosphoenolpyruvate carboxylase (PEPC) works as a primary carboxylase in the cytoplasm, forming oxaloacetate (C4) from phosphoenolpyruvate (C3) and HCO_3_^–^ (Figure 1B). This C4 acid is then transported into the chloroplast and releases CO_2_ in the vicinity of Rubisco by action of the enzyme phosphoenolpyruvate carboxykinase (PEPCK) (Reinfelder et al., 2000, 2004; Roberts et al., 2007a,b; and references therein). The process of decarboxylation can also be performed by the malic enzyme (ME). Oxaloacetate (OAA) is converted to malate via malate dehydrogenase which is then transferred to another compartment (likely mitochondria) and forms pyruvate and CO_2_ via ME (Kustka et al., 2014). Co-occurrences of PEPCK and ME driven decarboxylation mechanisms have been reported in C4 plants (Cacefo et al., 2019) and marine diatoms (Kroth et al., 2008). It has been proposed that ME may not be actively involved in CCMs and probably plays a role in photorespiration and mitochondrial metabolism in marine diatoms (Davis et al., 2017). The study by Kroth et al. (2008) stated that in case of a C4 pathway in diatoms, the processes of decarboxylation of OAA as well as malate and carboxylation by Rubisco may take place separately in mitochondria and plastids, respectively. In such a case the CO_2_ molecule released in mitochondria via decarboxylation needs to be transferred to Rubisco. It is possible that the CO_2_ is then converted to HCO_3_^–^ again via CA and a further conversion to CO_2_ takes place within the plastid in the vicinity of Rubisco before carboxylation. These double conversions involve a considerable amount of energy and diatoms may use a C4 CCM for dissipating extra energy which they acquire via the light reactions. Thus, diatoms living under optimum light conditions might actively use a C4 CCM, whereas the diatoms from light limited areas and in deep chlorophyll maxima may down-regulate this process to avoid energy loss.

However, the existence of a fully functional biochemical CCM (C4 pathway) in marine diatoms is not yet proven (Tanaka et al., 2014; Clement et al., 2016) despite some experimental studies (Roberts et al., 2007b; Kustka et al., 2014). A short term metabolic C^14^ labeling study of two model marine diatoms (*Thalassiosira pseudonana* and *Thalassiosira weissflogii*) showed that the initial labeled products in *T*. *pseudonana* were mostly C3 and C6, whereas *T. weissflogii* produced a mixture of C3 and C4 acids (Roberts et al., 2007b). Notwithstanding, C4 enzymes were documented in both species. This suggests that some diatoms may operate a mixture of C3 and C4 CCMs. A significant increase in expression of genes encoding C4 enzymes under low CO_2_ acclimatized cells was reported in model marine diatoms (Kroth et al., 2008; Saade and Bowler, 2009). However, the evidence for an active biochemical CCM in natural communities of marine diatoms has remained inconclusive.

Another inefficient feature of Rubisco in green algae and land plants is its deactivation by sugar phosphates (ribulose-1,5-bisphosphate and others). To perform optimum photosynthesis, Rubisco is usually reactivated by a motor protein, named Rubisco activase (RCA), by binding to the inactive Rubisco via ATP hydrolysis (Shivhare and Mueller-Cajar, 2017; Figure 1C). The gradient of pH and Mg^++^ concentrations are two key factors that control RCA activity. A non-substrate CO_2_ and a Mg^++^ ion need to bind to Rubisco before carboxylation and therefore the concentration of CO_2_ is also important for activation of Rubisco prior to carboxylation (Pollock et al., 2003). In the study by Young et al. (2016) it was noticed that the activation levels of Rubisco in eleven experimental diatom species were quite low suggesting a strong possibility for the presence of a RCA type of enzyme. Surprisingly, no structural homolog of RCA has been reported in diatoms. Instead, a functional homolog of RCA, denoted Calvin-Benson-Bassham protein (CbbX) complex, was identified from a red type Rubisco in proteobacteria (Mueller-Cajar et al., 2011) and red algae (Loganathan et al., 2016). Jensen et al. (2017) reported a BLAST search that revealed the presence of CbbX homologs in almost 100 stramenopiles including some model diatoms. The authors also established that CbbX is encoded in the plastid genome unlike in green plants where the *RCA* gene is encoded in the nucleus. However, it was subsequently found that in red algae and diatoms, another CbbX gene is also encoded in the nucleus (Bhat et al., 2017). Jensen et al. (2017) also argued in favor of the existence of an allosteric control of Rubisco by CbbX in diatoms (Figure 1C). Other than this, to our knowledge there have been no other studies of the abundance and functioning of CbbX in diatoms, neither in lab studies nor in natural populations. Conversely, only a few discrete studies have reported gene expression within phytoplankton communities as a function of changing ocean carbon chemistry (Endo et al., 2015; Hennon et al., 2015; Hopkinson et al., 2016). Moreover, most of the studies are based on model diatoms and hence there is a strong need to study natural diatom assemblages.

**FIGURE 2:**
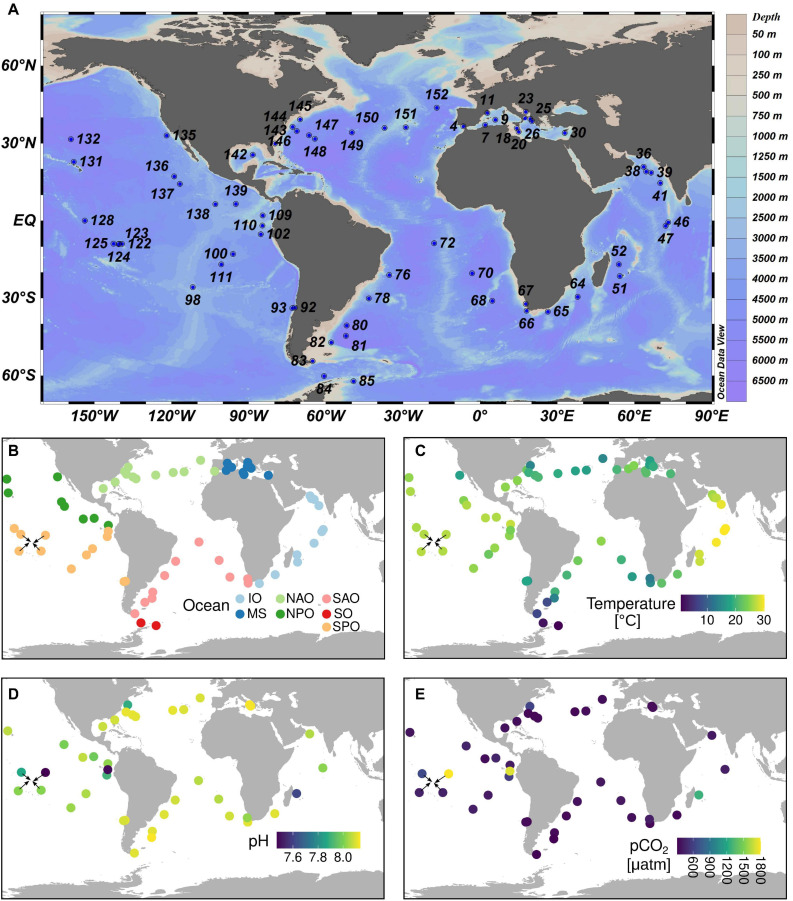
*Tara* Oceans sampling sites relevant to the current study. **(A)** Station labels. **(B)** Ocean regions. **(C)** Temperature measurements. **(D)** pH measurements. **(E)** CO_2_ partial pressure measurements. The sampling covers almost all main ecogeographic locations. Complete contextual data is available in Supplementary Table 1. IO, Indian Ocean; MS, Mediterranean Sea; NAO, North Atlantic Ocean; NPO, North Pacific Ocean; SAO, South Atlantic Ocean; SO, Southern Ocean; SPO, South Pacific Ocean.

Therefore, we deemed it important to characterize the diatom CCM in the environment under natural CO_2_ variability. With this motivation, the present study investigates the interlink between the abundance and expression of the genes encoding five key enzymes (CbbX, CA, PEPC, PEPCK, and ME) involved in diatom CCMs under variable CO_2_ levels. We did so by mining the *Tara* Oceans datasets (Figure 2), which were generated from samples across the global ocean in a standardized manner, including the measurement of carbonate chemistry and other physicochemical parameters and the generation of >200 metagenomes and >220 metatranscriptomes (Carradec et al., 2018).

## Materials and Methods

### Sequence Search and Analysis in the *Tara* Oceans Eukaryotic Gene Catalog

We searched for sequences of interest in version 1 of the Marine Atlas of *Tara* Oceans Unigenes (MATOU.v1; Carradec et al., 2018). It consists of 116 million transcribed sequences mainly from eukaryotic plankton in size fractions ranging from 0.8 to 2,000 μm. It was generated by assembling 441 poly-A + metatranscriptomes from samples across the main ocean basins (with the exception of the Arctic Ocean) and then clustered at 95% identity to define a non-redundant catalog (Carradec et al., 2018).

A HMMer search (version 3.2.1 with gathering threshold option)^1^ was performed in the translated version of MATOU.v1 using the following Pfam models: PF00004 (AAA; ATPase family associated with various cellular activities) for detecting CbbX, PF00311 (PEPcase; Phosphoenolpyruvate carboxylase) for PEPC, PF01293 (PEPCK_ATP; Phosphoenolpyruvate carboxykinase) for PEPCK, PF00390 (malic; Malic enzyme N-terminal domain) and PF03949 (Malic_M; Malic enzyme NAD binding domain) for ME, PF00194 (Carb_anhydrase; Eukaryotic-type carbonic anhydrase) for alpha-CA, PF00484 (Pro_CA; Carbonic anhydrase) for beta-CA, PF00132 (Hexapep; Bacterial transferase hexapeptide) for gamma-CA, PF10563 (CA_like; Putative carbonic anhydrase) for delta-CA, PF18484 (CDCA; Cadmium carbonic anhydrase repeat) for zeta-CA, PF18599 (LCIB_C_CA; Limiting CO_2_-inducible proteins B/C beta carbonic anhydrases) for theta-CA, and PF08332 (CaMKII_AD; Calcium/calmodulin dependent protein kinase II association domain) for iota-CA. To compare with primary and housekeeping pathways, we also retrieved the sequences coding for the nuclear-encoded subunits of photosystem II (PF05151, PsbM; PF01716, MSP; PF05757, PsbQ; PF06514, PsbU; PF18240, PSII_Pbs31) and for ribosomal proteins (112 Pfam models listed in Supplementary Table 1). Taxonomic assignment of MATOU.v1 is already available based on sequence similarity against a reference database containing UniRef90, MMETSP, and other sources (see Carradec et al., 2018). Based on this assignment, we only kept with those sequences assigned as diatoms for further analysis.

In order to discard homologous proteins of interest, we carried out a combination of sequence similarity network and phylogeny approaches for functional assignment. Briefly, we carried out a HMMer v3.2.1 search (as previously mentioned) for sequences containing the Pfam domains of interest among the sequenced genomes available in the Integrated Microbial Genome (IMG) database^2^ (Chen et al., 2018) and the sequenced transcriptomes from MMETSP (Keeling et al., 2014). The retrieved sequences were translated in the correct frame and the Pfam domain region was extracted. These sequences were used for building a protein similarity network using EFI-EST tool (Zallot et al., 2019) and Cytoscape visualization (Shannon et al., 2003), which allowed us to inspect the different protein clusters varying the score cut-off. By this step, we found that most Pfams were specific to the enzymes of interest (at least in diatoms) with the only exceptions of CbbX and iota CA (Supplementary Figures 1, 2). The final list of MATOU.v1 sequences used in the current work is displayed in Supplementary Table 1.

In the case of CbbX, it is part of one of the many clusters detected in the sequence similarity network of the AAA domain sequences (Supplementary Figure 1A). This network was built using a score cut-off of 40 after a previous step of reducing sequence redundancy to 80% identity with CD-HIT version 4.6.4 (Li and Godzik, 2006). Therefore, we then built a phylogeny for all the AAA domain sequences of this cluster (Supplementary Figures 1B,C). For this, we aligned the sequences with MAFFT version 6 using the G-INS-I strategy (Katoh and Toh, 2008) and used the resulting alignment to generate the tree with PhyML version 3.0 (Guindon et al., 2010). Four categories of rate variation were used. The starting tree was a BIONJ tree and the type of tree improvement was subtree pruning and regrafting. Branch support was calculated using the approximate likelihood ratio test (aLRT) with a Shimodaira–Hasegawa-like (SH-like) procedure. CbbX sequences formed a distinctive branch (Supplementary Figure 1B), which included the experimentally validated sequences from the proteobacterium *Rhodobacter sphaeroides* and the nuclear- and plastid-encoded versions from the red alga *Cyanidioschyzon merolae* (Loganathan et al., 2016). The remaining branches of the tree are annotated as stage V sporulation protein K (KEEG id: K06413) by BlastKOALA (Kanehisa et al., 2016). Therefore, the sequence similarity network and the phylogenies were used as references for the selection of *Tara* Oceans unigenes coding for diatom CbbX.

In the case of iota-CA, it forms one of the two main clusters in the sequence similarity network of CaMKII_AD domain sequences (Supplementary Figure 2), which was built using a score cut-off of 18 and a previous step of reducing redundancy at 90% identity with CD-HIT version 4.6.4 (Li and Godzik, 2006). The iota-CA cluster contains sequences from bacteria and eukaryotes, including the experimentally validated iota-CA from *T. pseudonana* (Jensen et al., 2019) as well as orthologous sequences from other species (Jensen et al., 2019; Nonoyama et al., 2019). The other subfamily contains eukaryotic sequences annotated as canonical Calcium/calmodulin dependent protein kinases. Therefore, we used the protein similarity network to keep exclusively with the iota-CAs among those MATOU-v1 sequences with the CaMKII_AD domain.

### Analysis of Biogeographical and Environmental Patterns of Gene and Transcript Abundances

*Tara* Oceans performed a worldwide sampling of plankton between 2009 and 2013 (Figure 2 and Supplementary Table 2) using a serial filtration system for separating the plankton into discrete size fractions (Pesant et al., 2015). In the current work, we analyzed a total of 203 metagenomes and 224 metatranscriptomes generated from samples of the surface layer (5 m depth) of 66 globally distributed stations (Figure 2) and corresponding to the four main size fractions enriched in protists: 0.8–5 μm, 5–20 μm, 20–180 μm, and 180–2,000 μm (Carradec et al., 2018). Thus, we retrieved the metagenomic and metatranscriptomic read abundances of the selected MATOU.v1 sequences (described in the section “Sequence Search and Analysis in the *Tara* Oceans Eukaryotic Gene Catalog”) and normalized them by the total read abundance for genes or transcripts of the whole diatom community of the corresponding sample. Results are displayed in Supplementary Table 3.

We compared the metagenomic and metatranscriptomic abundance patterns with the environmental data collected during *Tara* Oceans expeditions.^3^ The contextual data used in the current work is displayed in Supplementary Table 2. Carbonate chemistry was determined in 40 stations.^4^ Total alkalinity and DIC were measured potentiometrically (Edmond, 1970), and other carbonate chemistry parameters (pH on total scale, CO_2_, pCO_2_, HCO_3_^–^, CO_3_^–^) were calculated using seacarb (Nisumaa et al., 2010). The average CO_2_ values were 12 ± 2μmol kg^–1^ which is very common for present day surface seawater values. However, there were four stations (TARA_110, TARA_122, TARA_052, TARA_145) with more than double these CO_2_ levels. The station locations were highly diverse; from tropical, subtropical and higher latitude locations (from 54.37°S to 43.67°N) including upwelling, shallow lagoon and deep sea stations (Figure 2 and Supplementary Table 2).

Measurements of temperature, conductivity, salinity, depth, pressure, and oxygen were carried out at each station with a vertical profile sampling system (CTD-rosette) and Niskin bottles (Picheral et al., 2014). Chlorophyll *a* concentrations were measured using high-performance liquid chromatography (Van Heukelem and Thomas, 2001; Ras et al., 2008). Phosphate and silicate concentrations were determined using segmented flow analysis (Aminot et al., 2009). Iron concentrations were derived from the biogeochemical model PISCES2 (Aumont et al., 2015). Monthly average estimates of photosynthetically active radiation (PAR) were derived from satellite data^5^.

### Plotting and Statistical Analysis

All analyses were carried out in R language^6^. Correlation matrices were generated with the *rcorr* function of the *Hmisc* package and plotted using the *corrplot* library. Other graphs were plotted with R library *ggplot2* (Wickham, 2009). Spearman rho correlation analysis were carried out with *cor.test* function.

## Results

### Diversity and Abundance of Sequences Coding for Diatom Carbon Dioxide Concentration Enzymes

To investigate the diversity and environmental distribution of CCMs in natural populations of diatoms, we searched for sequences coding for CbbX, CA, PEPC, PEPCK, and ME in the eukaryotic unigene catalog of *Tara* Oceans (Carradec et al., 2018) using profile hidden Markov models and sequence similarity networks (see section “Materials and Methods”). The total number of retrieved distinct diatom sequences was: 40 for CbbX, 4,860 for CAs, 943 for PEPC, 488 for PEPCK, and 336 for ME. The obtained CA sequences corresponded to the following classes: 434 alpha, 39 beta, 1,231 delta, 895 gamma, 1,477 iota, 637 theta, and 147 zeta (Supplementary Table 1).

**FIGURE 3:**
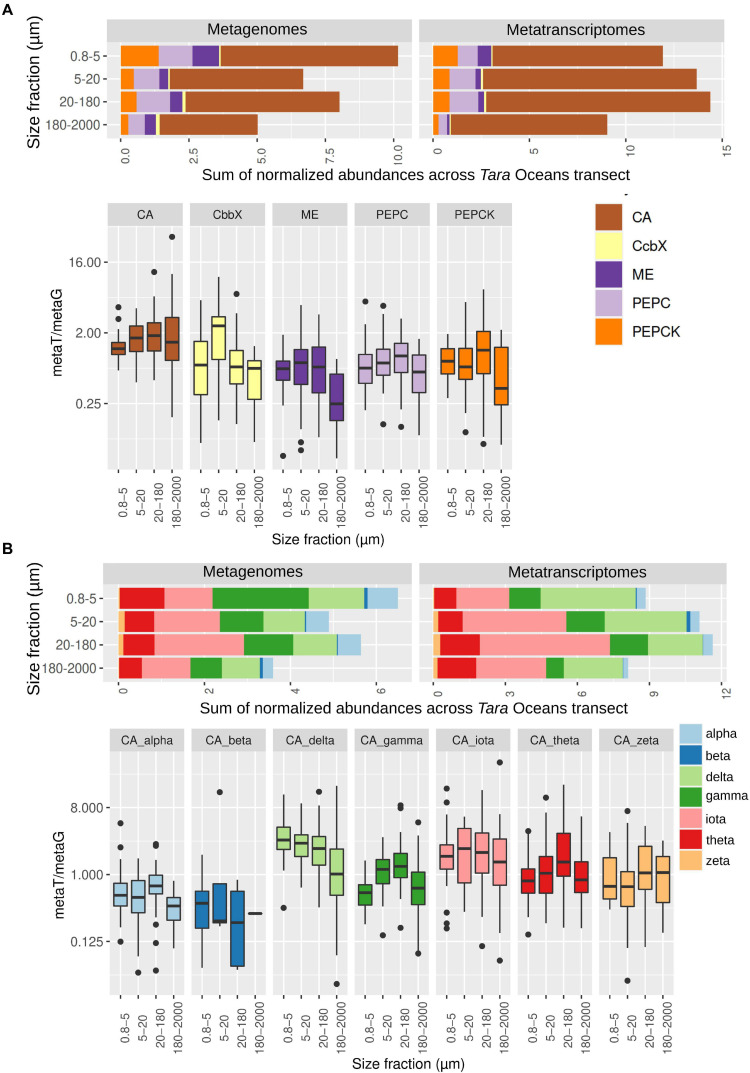
Abundance of genes and transcripts potentially involved in diatom carbon dioxide concentration mechanisms across the different size-fractionated seawater samples collected during the *Tara* Oceans transect. **(A)** Gene and transcript abundances for the five enzymes under study. Barplots show the sum of normalized abundances for all samples in a given size fraction. Boxplots show the gene expression levels based on the abundance ratio in metatranscriptomes and metagenomes (metaT/metaG), and are displayed in logarithmic scale. Abbreviations: carbonic anhydrase (CA), Rubisco activase (CbbX), malic enzyme (ME), phosphoenolpyruvate carboxylase (PEPC), and phosphoenolpyruvate carboxykinase (PEPCK). **(B)** Gene and transcript abundances for the different types of CAs. Barplots and boxplots are displayed as indicated in panel **(A)**.

We then retrieved the metagenomic and metatranscriptomic read abundances of these sequences across the four main eukaryotic size fractions (Figure 3A and Supplementary Figure 3 and Supplementary Table 3). CAs were dominant both in gene number and transcript abundance, with almost one order of magnitude higher levels than the other enzymes under study (Figure 3A). CAs comprise on average the 0.2% of the total diatom metatranscriptomic reads, which is similar to the values of all nuclear-encoded subunits of photosystem II (Supplementary Figure 3B). These results emphasize the importance of CAs in diatom CCMs. For the five enzymes, we found differences between size fractions, probably related with differential needs for maintaining CCMs according to cell sizes and/or aggregation forms: while CbbX gene and transcript abundance increases when moving toward the bigger size fractions, the opposite is observed for the other enzymes (Figure 3A and Supplementary Figure 3).

Among the different classes of CAs (Figure 3B and Supplementary Figure 4), delta, gamma and iota are the most abundant (18–37% and 9–47% of the total CA gene and transcript abundance, respectively, with the percentage range corresponding to the minimum and maximum values depending on the size fraction), followed by theta (13–16% and 9–10%) and alpha (7–11% and 2–4%), whereas zeta and beta represent <2% of gene or transcript abundance. Iota-CA showed the highest gene abundances, and the highest transcript abundances together with delta-CA. The CA classes show differences in abundance between metagenomes and metatranscriptomes, reflecting differences in the expression levels of their genes (Figure 3B). Delta CA is the most expressed and shows a clear expression increase toward the smaller size classes. It is followed by iota, whose expression does not vary between size fractions. On the opposite, alpha and beta are the least expressed classes.

**FIGURE 4:**
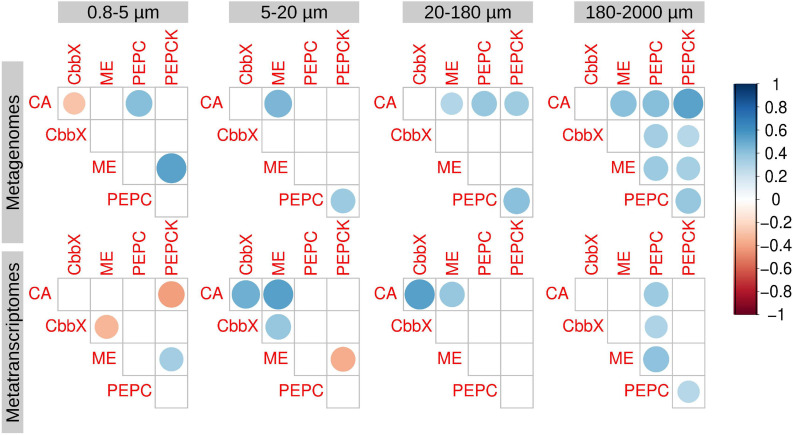
Correlation analysis between the diatom genes and transcripts potentially involved in carbon dioxide concentration mechanisms. Circle size and color intensity are proportional to the Spearman’s rho correlation coefficients. Empty spaces refer to non-significant correlation values (two.tailed *p*-value > 0.05). carbonic anhydrase (CA), Rubisco activase (CbbX), malic enzyme (ME), phosphoenolpyruvate carboxylase (PEPC), and phosphoenolpyruvate carboxykinase (PEPCK).

We also analyzed the correlations between the transcript abundances of the different enzymes (Figure 4). In general, we did not find strong correlations in expression of the potential components of a biochemical CCM: ME, PEPC, and PEPCK (Figure 4). An exception was nonetheless noted in the largest size fraction (180–2,000 μm) (Figure 4), where epizoic and large chain-forming diatoms are found. Thus, this pathway cannot be discarded, but it seems clear that it would not be universal in diatom communities.

### Biogeographical Distribution of Genes and Transcripts of Diatom Carbon Dioxide Concentration Enzymes Show Abundance Hotspots

**FIGURE 5:**
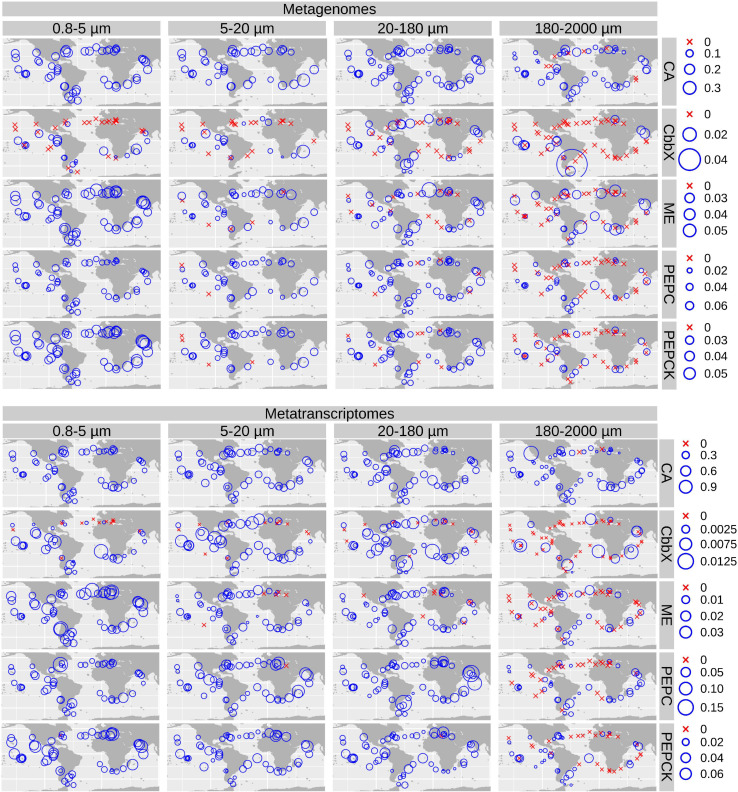
Biogeography of genes and transcripts potentially involved in diatom carbon dioxide concentration mechanisms. Circle sizes are proportional to the gene or transcript abundance (% of total diatom gene or transcript read abundance), while crosses indicate absence of detection. Color code varies according to the size fractions. CA, carbonic anhydrase; CbbX, Rubisco activase; ME, malic enzyme; PEPC, phosphoenolpyruvate carboxylase, PEPCK, phosphoenolpyruvate carboxykinase.

We plotted the biogeographical abundance distributions of the genes and transcripts under study (Figure 5 and Supplementary Figures 5, 6). All enzymes show a widespread occurrence, but with some regional patterns in abundance. A clear regional pattern is found for PEPCK, which shows its lowest gene and transcript abundances in the Southern Ocean (SO) across all size fractions. In addition, we detected several stations that can be considered abundance hotspots for the genes and/or the transcripts coding for carbon concentrating enzymes, but showing divergence between size fractions, pointing to the effect of cell size and/or aggregation.

**FIGURE 6:**
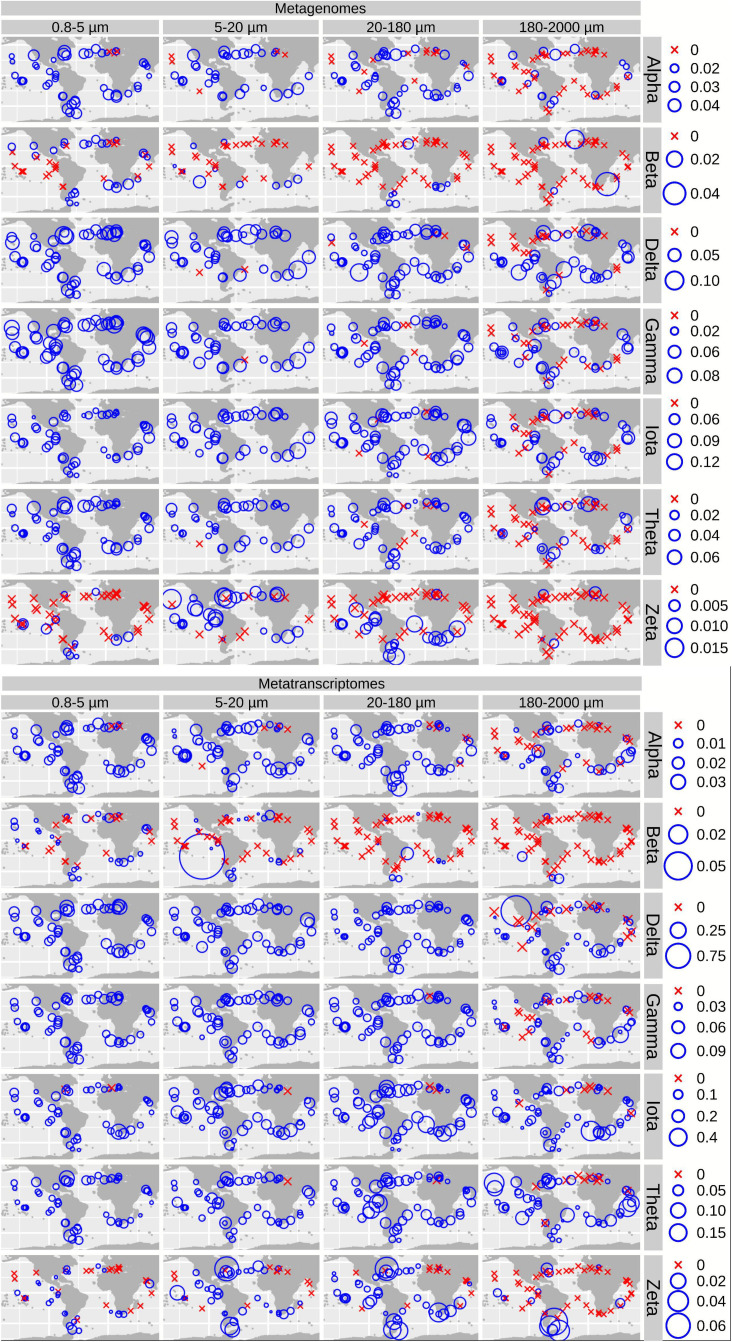
Biogeographical patterns for the diatom genes and transcripts encoding carbonic anhydrases. Circle sizes are proportional to the gene or transcript abundance (% of total diatom gene or transcript abundance), while crosses indicate absence of detection.

For CA, the highest gene and transcript abundances were detected in the Indian and North Atlantic Oceans (IO and NAO, respectively) as well as in a few stations in the South Atlantic Ocean (SAO; Figure 6 and Supplementary Figures 7, 8). The most abundant CA classes are widespread in the global ocean (but with some differences in their abundances). On the contrary, the low-abundant zeta and beta classes are mainly detected outside the equatorial region (Figure 6).

### Correlations Between the Environmental Variables and Genes Encoding Diatom Carbon Dioxide Concentration Enzymes

**FIGURE 7:**
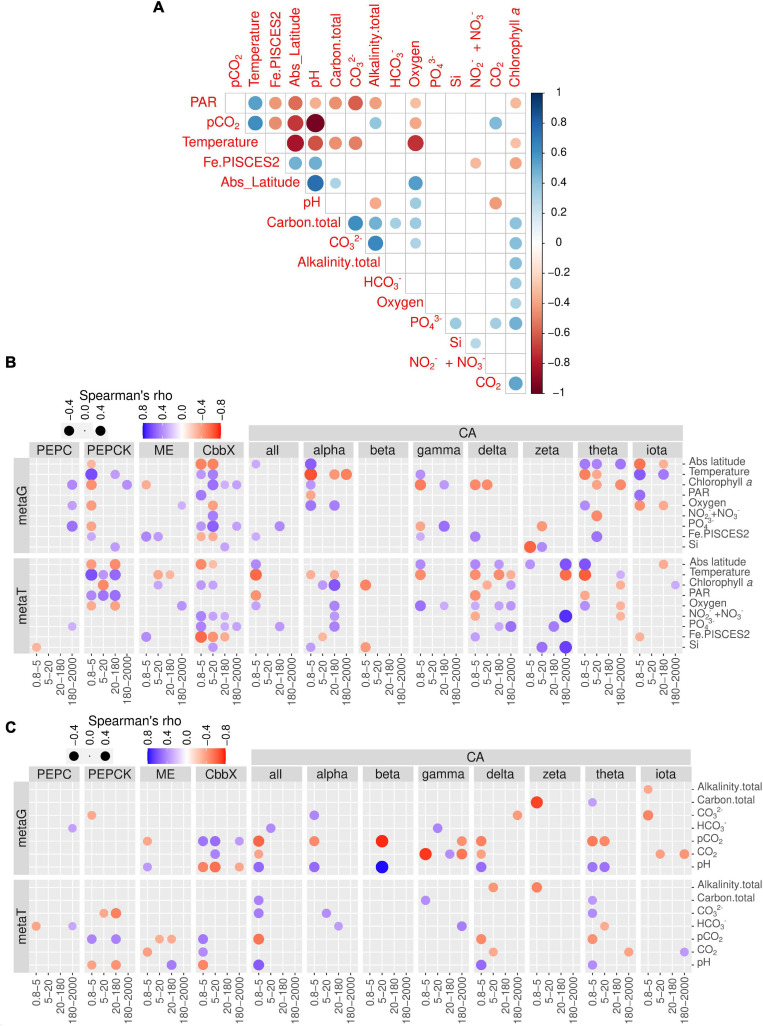
Environmental distribution of genes and transcript potentially involved in diatom carbon dioxide concentration mechanisms. **(A)** Pairwise correlation of the matrix of contextual parameters. **(B)** Correlations of nutrients, chlorophyll *a* and temperature with gene and transcript abundances for the enzymes under study. **(C)** Correlations of carbonate chemistry measurements with gene and transcript abundances for the enzymes under study. Circle color varies according to Spearman rho’s correlation coefficient, while size varies according to the absolute value of the coefficient. Only statistically significant correlations are displayed (two-tailed test, *p* < 0.05). PAR, photosynthetically active radiation; CA, carbonic anhydrase; CbbX, Rubisco activase; ME, malic enzyme; PEPC, phosphoenolpyruvate carboxylase, PEPCK, phosphoenolpyruvate carboxykinase.

We carried out a correlation analysis between gene and transcript abundances of the enzymes under study and the physicochemical and carbon chemistry variables (Figure 7). Many of these variables are correlated among each other (Figure 7A), which has to be taken into account when interpreting the patterns.

When focusing on transcript abundances, PEPCK and CbbX displayed an anticorrelation with absolute latitude, whereas ME and most of the CA classes showed the opposite (Figure 7B). These patterns can be related to the effect of temperature and/or PAR, or the fact that in the current dataset the absolute latitude is linked to nutrient and carbon chemistry variables (Figure 7A). CbbX is correlated with phosphate, as are many CA classes.

The correlation matrix with the carbon chemistry variables and CCM enzymes are displayed in Figure 7C. The trends revealed that the partial pressure of CO_2_ displayed no correlation with PEPC transcript abundance in any size fraction. By contrast, PEPCK showed strong positive correlations in two size fractions (0.8–5 and 20–180 μm) with the partial pressure of CO_2_ and strong negative correlations with pH. On the contrary, ME was significantly negatively correlated with CO_2_ and positively with pH. Interestingly, CbbX, the least expressed enzyme of diatom CCM, showed significant positive correlations with CO_2_ (partial pressure and concentrations) and negatively varied with pH only in the smallest size fractions.

CAs in general displayed strong positive correlations with bicarbonate, carbonate ion concentrations, as well as total alkalinity, and negatively correlated with the partial pressure of CO_2_ only in the smallest size class. Specifically, delta and theta classes show strong negative correlations with the partial pressure of CO_2_ for smaller size groups. Surprisingly, iota-CA, one of the most abundant CAs, was generally not well correlated with the carbon chemistry variables. Similarly, beta, gamma and zeta-CA did not show any clear trends with carbon chemistry parameters. Zeta-CA gene expression levels for the largest size class exhibited strong positive correlations with absolute latitude, Si and NO_3_- + NO_2_- levels and varied inversely with temperature (Figure 7B). The expression levels of alpha, delta, gama, and theta for the smallest size class were negatively correlated with temperature and hence the average expression level for all CAs also indicated a similar trend.

## Discussion

### CbbX

The identification of CbbX and its functional role as a Rubisco activation system in diatoms were reported less than a decade ago (Mueller-Cajar et al., 2011) and very little information is available from natural diatom assemblages. We present here the first baseline data regarding the natural variability of this important protein.

The number of CbbX sequences was very low compared with the other sequences. This can be related to the fact that the *Tara* Oceans gene catalog corresponds to assembled sequences from transcriptomes of polyadenylated RNA (Alberti et al., 2017; Carradec et al., 2018), thus minimizing the detection of plastid-encoded versions of CbbX. In addition chloroplast sequences were removed from the final catalog (Carradec et al., 2018), which might also filter the nuclear-encoded versions of CbbX due to its similarity to the plastid encoded versions (Bhat et al., 2017).

The metatranscriptomic read abundance for the sequences coding for CbbX was also very low. *A priori*, a high expression would be expected if we consider the ability of marine diatoms to fix one fifth of global carbon fixation per year and that Rubisco is the most abundant protein on the planet. However, this low total metatranscriptomic read abundance is probably an underestimation due to the low number of retrieved sequences, as the expression of these genes (based on the abundance ratio between metranscriptomes and metagenomes) is similar to those of the other enzymes under study (Figure 3A). In addition, low transcript abundances do not necessarily imply a low enzymatic activity. It can be possible that the CbbX function in marine diatoms is controlled by both nuclear and plastid-encoded CbbX versions. Moreover, the gene expression for both CbbX and Rubisco can be linearly varied, and hence a low transcript abundance for CbbX would indicate low transcript abundance for Rubisco. Indeed, diatoms possess an efficient CCM, thus they do not require a high Rubisco concentration: the amount is <6% of the total cellular protein according to both field and culture experiments (Losh et al., 2013), much less than in land plants. All this information may justify the low transcript levels for CbbX in the current work. This must be particularly true in the oligotrophic open ocean where nitrogen can be limiting because Rubisco plays a role as a nitrogen reservoir (Herrig and Falkowski, 1989). Under nitrogen limitation, the nuclear-encoded proteins are synthesized preferentially over those proteins that are encoded in the plastid (Herrig and Falkowski, 1989). In this contest, it is worth mentioning the significant positive correlation between transcript levels for CbbX and NO_3_^–^ + NO_2_^–^ concentrations. The strongest correlation was observed for the smallest size fraction while no correlation was detected for the largest size range, which can be related to the fact that smaller diatoms allocate lesser nitrogen resources to build Rubisco than the large centric diatoms (Wu et al., 2014). Finally, the negative correlation of gene and transcript abundance for CbbX against Fe concentrations may indicate its higher activity in the open ocean iron-limited areas. Under nitrogen limitation, the cellular demand for Fe can be significantly low since Fe is essential for nitrogen metabolism. Small-sized diatoms growing under low nitrogen and Fe limited area can allocate less amount of nitrogen resource to synthesize Rubisco and this could be an evolutionary strategy for the open ocean diatoms.

Our analysis shows that the gene abundance and expression levels of CbbX were positively correlated with pCO_2_ and negatively related with pH in the smallest size fraction (0.8–5 μm). This trend suggests that within the smallest diatoms from the global ocean, CbbX activation is likely to be a prominent feature. CbbX homologs have been detected in the model diatoms *T. pseudonana* and *Phaeodactylum tricornutum*, as well as *Asterionella formosa* and other stramenopiles (Jensen et al., 2017). Nevertheless, it has been already shown that the quantitative level of Rubisco protein does not represent the rate of carboxylation (Raines, 2003; Gontero and Salvucci, 2014). This is likely because the rate of carboxylation is controlled principally through the activation of Rubisco by a motor protein like CbbX (Jensen et al., 2017). However, such a conformational change of Rubisco from an inactive protein to its active form involves several factors and is more complicated than was initially presumed (Gontero and Salvucci, 2014). Therefore, our observation reveals the likely significance of CbbX protein in diatoms. Furthermore, the structure and activation mechanism of RCA in higher plants or green algal lineages are considerably different from the CbbX protein found in red algal lineage taxa characterized by ID type Rubisco. Based on such information, it has been hypothesized that these two different types of motor proteins for Rubisco activation probably resulted from convergent evolution coupled with changing atmospheric CO_2_/O_2_ levels (Mueller-Cajar et al., 2014).

Based on the recently reported high diversity of diatom CCMs (Young et al., 2016; Iñiguez et al., 2020) and the efficiency of the Rubisco 1D type, it has been postulated that diatom CCMs and Rubisco might have co-evolved (Young and Hopkinson, 2017) with changing environmental variables like decreasing CO_2_ and increasing O_2_ levels (Reinfelder, 2011; Clement et al., 2017). The photorespiratory energy loss is relatively lower in marine diatoms than in other phytoplankton (Rech et al., 2008), while the specificity factor (τ) of Rubisco for CO_2_ relative to O_2_ is considerably higher (Tortell, 2000). This strengthens the fact that diatoms are capable of maintaining a high CO_2_:O_2_ ratio in the vicinity of Rubisco through active DIC pumping systems (Reinfelder, 2011). The main evolutionary diversification in marine diatoms took place during the time when atmospheric CO_2_ levels dropped significantly (Reinfelder et al., 2000) and therefore diatoms among the other phytoplankton groups are likely to have developed the most efficient CCMs and Rubisco type (Young et al., 2012). This type ID Rubisco from red algal lineage can perform its highest activity under low CO_2_:O_2_ ratio and demands low nutrients as well as energy investment in a CCM; this was likely to be the key factor for mass expansion of diatoms and coccolithophores in the Phanerozoic oceans under very high O_2_ and low CO_2_ levels (Rickaby and Hubbard, 2019).

At the heart of the CCM of diatoms and other algae is the pyrenoid (Badger et al., 1998), a spherical structure in the chloroplast stroma consisting of a matrix of tightly packed Rubisco and RCA. The molecular mechanism by which Rubisco aggregates to form the pyrenoid matrix was recently resolved in the model green alga *Chlamydomonas reinhardtii*, where a low-complexity repeat protein, Essential Pyrenoid Component 1 (EPYC1), links Rubisco to form the pyrenoid (Mackinder et al., 2016). The primary sequences of disordered proteins like EPYC1 are known to evolve rapidly compared with those of structured proteins, but their physicochemical properties are under selective pressure and are evolutionarily maintained. Therefore, Mackinder et al., 2016 searched for proteins with similar physicochemical properties (i.e., repeat number, length, high isoelectric point, disorder profile, and absence of transmembrane domains) across a broad range of algae. They found potential EPYC1-like proteins in the diatoms *T. pseudonana* and *P. tricornutum*, which do not exhibit sequence conservation between them. Expectelly, a BLAST search using these sequences against the MATOU-v1 catalog did not retrieve any similar sequences (data not shown).

### Carbonic Anhydrases

Carbonic anhydrases are one of the highest upregulated CCM enzymes in diatom cells grown in CO_2_ limited conditions (Clement et al., 2017), however, CAs also play several other physiological roles apart from photosynthesis (Raven, 1995). Out of eight different types of CAs (Jensen et al., 2020), seven subclasses of CAs are found to be constitutively expressed in diatoms (Samukawa et al., 2014; Jensen et al., 2019). The present study also noticed the presence of the expressed genes of all eight types of CAs in the natural diatom populations from the surface ocean. Such high variability and abundance of CAs in diatoms are quite exclusive relative to other organisms and could be due to their evolutionary complexity. The fact that CA transcript levels are the highest in the *Tara* Oceans dataset also explains its profound role in CCMs in marine natural populations of diatoms and indicates that diatoms in the global oceans are likely to be operating a biophysical CCM. Marine diatoms usually show very high intercellular conversion of bicarbonate to CO_2_ and vice-versa to maximize CO_2_ levels in the vicinity of Rubisco and reduce the diffusive loss of CO_2_ from the cell (Matsuda and Kroth, 2014) and hence the significance of CAs are eminently important. Zeng et al. (2019) noticed a strong correlation between Rubisco and CA activities in the model marine diatom *P. tricornutum* and suggests that the rate of carboxylation is directly dependent on the rate of DIC supply which is mediated by CA.

The subcellular location of different CAs can be directly linked to CO_2_ acquisition. There are some isoforms which are found in the diatom chloroplast, such as iota-CA, beta-CA and theta-CA (Tanaka et al., 2005; Kikutani et al., 2016). The proximity of such CAs to Rubisco probably results in a more efficient CO_2_ acquisition. Consistent with this view, our observation of a significant negative correlation between gene and transcript abundances of theta-CA against pCO_2_ for the smallest size fraction also points to an upregulated function of this enzyme at low pCO_2_ levels. The presence of the chloroplast-targeted theta-CA in some haptophyte species suggest that the diatom ancestor might have acquired this CA gene via horizontal gene transfer (Nonoyama et al., 2019). Regarding iota-CA, there are many gene copies coding for chloroplast-targeted iota-CAs in common marine diatoms like Odontella, but in a few other species the gene is absent (Nonoyama et al., 2019).

The recent research by Clement et al. (2017) reported the regulation of the latest type of CA, known as “Low CO_2_ inducible protein of 63kDa” or LCIP63 in the marine diatom *T. pseudonana*. Later Jensen et al. (2019) confirmed its biochemical function as a CA and renamed it as iota-CA, also showing its widespread occurrence in the *Tara* Oceans dataset. Most importantly, the authors also reported that this type of CA showed its highest expression in surface waters and decreased with increasing depths. It should therefore also be noted that CCMs are likely to play a role in energy dissipation to remove extra energy from the cells and hence, under light inhibition in surface waters, CCMs involving this iota-CA might be used both for carbon acquisition as well as energy dissipation (Kroth et al., 2008). With increasing depth, light stress reduces and CO_2_ levels increase and therefore, the need for running a CCM involving iota-CA may be reduced. Our results also found that the highest abundant and expressed gene was iota-CA within marine diatoms from surface waters of the global ocean.

The absence of any significant correlation between iota-CA and carbon chemistry in general probably suggests that this enzyme functions despite CO_2_ variability in surface waters. This shows that the expression levels of CAs may not necessarily be coupled with CO_2_ levels. For example, in the coccolithophore *Emiliania huxleyi* the transcript of a delta-CA can exhibit high levels of expression irrespective of CO_2_ variability (Soto et al., 2006).

Carbonic anhydrase-zeta showed its highest expression at high latitudes for 180–2,000 μm size and seemed to be associated with larger diatoms. The positive correlations with NO_2_^–^ + NO_3_^–^ and Si levels also support this view since the large-celled diatoms in high latitude regions are usually found within eutrophic waters because they have very low surface area to volume ratios.

Within the smallest size fraction (0.8–5 μm) the positive correlation between CA gene expression and pH (coupled with negative correlation with pCO_2_) indicates that under high pH smaller diatoms use CA in their CCMs.

Our results also show that CAs are ubiquitous among all size classes of diatoms, and display high diversity. The abundance and expression of different types of CAs can largely be impacted by trace metal availability in the sea. Importantly, marine diatoms showed the ability to replace a specific metal ion with other more available forms under metal limited conditions (Lane et al., 2005). These metalloenzymes mostly use zinc (Zn) as a cofactor, but other metals such as cadmium (Cd), cobalt (Co), iron (Fe), and manganese (Mn) have also been reported to be associated with different CAs (Morel et al., 2020). In fact the Zn-CAs have been identified to substitute Zn with Co and Cd in surface waters (Morel et al., 2020). In the present study, out of these seven CAs detected, alpha, beta and theta-CAs use Zn ions, whereas, gama, delta and zeta showed the ability to substitute Zn with other metal ions including Cd, Co, and even Fe (Jensen et al., 2020). The highest expression (i.e., metatranscriptomic to metagenomic abundance ratios) were seen in those CAs which are capable of replacing Zn with other metals (Figure 3B). The recently identified iota-CA contains Mn and the availability of Mn can be much higher than Zn, particularly in coastal regions. Hence, marine diatoms might have selectively used this particular Mn-containing CA to cope with the available metal ions. However, this will remain a topic for future research to correlate different CA abundance and expression with trace metal concentrations in the global oceans.

### The C4 Enzymes

Our analyses revealed that the transcripts for the enzymes of the putative C4 biochemical CCM did not display co-occurring profiles, with the exeption of the largest size fraction (180–2,000 μm). It has to be noted that this size fraction has a prevalence of copepods, considered one of the most abundant multicellular organisms on the planet, and thus the sequencing signal from diatoms is weaker than in the other size fractions. This can be reflected by the higher variability in this size fraction with respect to the absence/presence of diatom genes and transcripts in the different sampling sites. Therefore, we cannot extend so far the speculations about this biochemical pathway, but it seems clear that the process is unlikely to be prevalent in natural communities, as the transcript levels for the three enzymes of a potential biochemical CCM were significantly lower than CA.

There are many experimental studies on marine diatoms showing the expression of all C4 enzymes (Reinfelder et al., 2000, 2004, Reinfelder, 2011; Roberts et al., 2007b), however their active functioning was not confirmed. The negative correlation between gene expression levels of ME and pCO_2_/fugacity (as well as the positive correlation with pH) suggests that under CO_2_ limitation the diatoms are likely to use this enzyme (except in the largest size fraction). Clement et al. (2017) observed that ME showed the lowest activity among all C4 enzymes and the ratio of Rubisco to PEPC was persistently >1 in the experimental marine diatoms. Our results are also consistent with this observation. Haimovich-Dayan et al. (2013) conducted an experiment by genetically silencing an essential C4 enzyme (pyruvate−orthophosphate dikinase, PPDK) in *P. tricornutum* and observed no major reduction in carboxylation rate. The authors concluded that marine diatoms are likely to use a C4 CCM for dissipating extra light energy. In another study by McGinn and Morel (2008), it was noticed that inhibition of two C4 enzymes (PEPC and PEPCK) resulted in significant reduction in photosynthetic activity in three model marine diatoms. There was almost no study available from any natural diatom population on this aspect and therefore, this study confirms that the relative contribution of C4 CCMs in surface water diatoms is significantly lower than C3 CCM. Moreover, a detailed investigation on deep chlorophyll maxima diatoms is essential to have a clearer picture about functioning of C4 CCM in marine diatoms. Furthermore, additional information on bicarbonate transporter proteins would also shed more light on this topic.

There is some experimental evidence showing higher resilience of phytoplankton communities to increasing CO_2_ levels from the oceanic region within the “subtropical north and above” (Schulz et al., 2013; Holding et al., 2015; Hoppe et al., 2017 and references therein). Diatoms from the Arctic and other high latitude seas showed high resilience to variable CO_2_ levels (Feng et al., 2009; Hoppe et al., 2018a,b; Sett et al., 2018; Wolf et al., 2018, 2019). This suggests that certain diatom species have high physiological plasticity to tackle the problem of increasing CO_2_ levels and therefore, no alteration in photosynthetic performance or growth rate was noticed in relation to changing CO_2_ levels in the experimental simulations (Hoppe et al., 2018a; Hoppe et al., 2018b; Wolf et al., 2018; Biswas et al., unpublished data). The diatoms from this region are likely to possess a constitutive CCM and therefore variable CO_2_ levels did not reveal any correlation with the gene expression of CbbX protein and other enzymes.

Hoppe et al. (2018a, b) and Wolf et al. (2018), and Biswas et al. (unpublished data) showed that Arctic diatoms are also highly resilient to the combined stress of irradiance and CO_2_ levels. This suggests that they have highly evolved cellular mechanisms to counteract photo-inhibition mechanisms. Unpublished data from Biswas et al. showed that an Arctic diatom has high plasticity to control pigment synthesis to combat light limitation/inhibition. Likewise, active functioning of CCM in the surface waters also could be used for these diatoms and the expression levels of C4 enzymes as well as CA can be high. Low latitude phytoplankton may face a stronger impact of photo-inhibition, particularly in the surface waters than the high latitude groups (Tortell, 2000). Hence, the cells living in surface waters may trade off cellular energy between photo-protection and carboxylation. In that case, CA gene expression may be high on the surface. Light is never limiting in this region and hence light dependent DIC uptake can never be hampered. In an experimental study by Biswas et al. (2017) on a tropical coastal diatom community, it was noticed that when light and CO_2_ both became limiting, carboxylation significantly hampered and resulted in low organic carbon accumulation. On the other hand, under saturated light the signature of non-photochemical quenching was noticed, even though carbon biomass accumulation was higher. Moreover, there is a continuous need of photosystem repair in the surface water due to the breakdown of the D1 protein of pigment system-II (Lavaud et al., 2016). A CCM, either C3- or C4-like mechanism could also be used for dissipating extra light energy in the surface waters (Haimovich-Dayan et al., 2013). It is also possible that a functional CCM in diatom cells from this region may help alleviate light stress and allow photosynthetic performance to remain unaffected. The recent study by Jensen et al. (2019) showed that iota-CA showed the highest expression in surface waters and decreased with increasing depth. Light/energy limitation in the subsurface water may be the reason for such down regulation (Kroth et al., 2008).

## Conclusion

This is the first attempt to assess the diversity, abundance, and distribution of CCMs in natural diatom assemblies at a global ocean scale. We carried out paired metagenomic and metatranscriptomic analyses, targeting five key enzymes, including components of the physical pathway as well as components associated with the putative biochemical mechanism.

We observed changes in transcript abundances in the different size fractions depending on the enzymes, pointing to the effect of different cell sizes and/or aggregation forms, such as chains.

CA was the most abundant and highly expressed gene with almost an order of magnitude higher values than the remaining enzymes, thus confirming the importance of biophysical CCM in natural diatom communities. Among the different classes of this enzyme, the most prevalent was the iota class, which was only recently characterized as a CA (Jensen et al., 2019) and so the information presented here represents the first data on its abundance in natural diatom assemblages.

Biogeographical and environmental distributions showed a complex pattern of responses to CO_2_ levels, total phytoplankton biomass, temperature and nutrient concentrations. This is in part due to the current limitations in the dataset, such as the correlations between different environmental variables or the poor representation of certain conditions. The future generation of data from new regions (e.g., Arctic Ocean) can ameliorate these limitations. It is nevertheless expected to obtain complex patterns when assessing the bulk responses of natural diatom populations, since species can differ in their physiological and molecular responses to the environment.

The transcript levels for the three enzymes of a potential biochemical CCM were significantly lower than CA. In addition, we did not find strong correlations among them, except in the largest size fraction (180–2,000 μm), where epizoic and large chain-forming diatoms are found. Thus, while the biochemical pathway cannot be excluded, it seems clear that the process is unlikely to be prevalent in natural communities.

Overall, this work provides a snapshot of diatom CCMs in the global ocean, providing valuable information toward the prediction of diatom responses in an ocean under anthropogenic change.

## Data Availability Statement

The original contributions presented in the study are included in the article/Supplementary Material, further inquiries can be directed to the corresponding author.

## Author Contributions

HB and CB designed the project. JJPK carried out the bioinformatic analysis. JJPK, CB, and HB analyzed the results and wrote the manuscript. All authors contributed to the article and approved the submitted version.

## Conflict of Interest

The authors declare that the research was conducted in the absence of any commercial or financial relationships that could be construed as a potential conflict of interest. The reviewer YM declared a past co-authorship with one of the authors CB to the handling editor.

## Footnotes

1 http://hmmer.org/

2 http://img.jgi.doe.gov

3 https://doi.org/10.1594/PANGAEA.875582

4 https://doi.pangaea.de/10.1594/PANGAEA.875567

5 https://modis.gsfc.nasa.gov/

6 http://www.r-project.org/

## Supplementary Material

Supplementary Figure 1 Sequence analysis of CbbX and homologs. **(A)** Protein similarity network for the Pfam domain AAA (PF00004). Each node represents a given sequence and those sequences with similarity higher than a score cutoff are linked (score cut-off of 40 in blast alignment). The network was built with sequences retrieved from the literature and from reference genomes and transcriptomes. Nodes are colored according to their taxonomy. The cluster containing CbbX sequences and close homologs is circled. **(B)** Phylogeny of the Pfam domain AAA from the sequences belonging to the cluster highlighted in panel **(A)**. The branch for CbbX is colored in yellow, whereas the remaining back branches are annotated as stage V sporulation protein K. **(C)** Phylogeny of the Pfam domain AAA from CbbX sequences, corresponding to the branch highlighted in panel **(B)**. Color code varies according to the taxonomy. The sequence similarity network and the phylogenies were used as references for the selection of *Tara* Oceans unigenes encoding diatom CcbX. The list of sequences and the alignment are available in Supplementary Table 1.

Supplementary Figure 2 Sequence analysis of iota carbonic anhydrase and homologs. Protein similarity network for the Pfam domain CaMKII_AD (PF08332). Each node represents a given sequence and those sequences with similarity higher than a score cutoff are linked (score cut-off of 18 in blast alignment). The network was built with sequences retrieved from the literature and from reference genomes and transcriptomes, as well as *Tara* Oceans unigenes. Nodes are colored according to their taxonomy. The cluster containing the iota carbonic anhydrase is encircled, as well as the cluster containing calcium/calmodulin-dependent protein kinase II. The list of sequences is available in Supplementary Table 1.

Supplementary Figure 3 Relative abundance of genes and transcripts potentially involved in diatom carbon dioxide concentration mechanisms in comparison to genes involved in other metabolisms. **(A)** Sum of normalized abundances for all samples in a given size fraction. **(B)** Gene and transcript abundances. Values in the box plots correspond to the% of total diatom gene or transcript abundance in the corresponding sample, and are displayed in log_2_ scale. In order to compare with other pathways, we also show the abundances for ribosomal proteins and for the nuclear-encoded subunits of photosystem II. Abbreviations: PSII, photosystem II; CA, carbonic anhydrase; CcbX, Rubisco activase; ME, malic enzyme; PEPC, phosphoenolpyruvate carboxylase; PEPCK, phosphoenolpyruvate carboxykinase.

Supplementary Figure 4 Relative abundance of genes and transcripts coding for the different classes of diatom carbonic anhydrases across the size-fractionated seawater samples collected during the *Tara* Oceans transect. **(A)** Sum of normalized abundances for all samples in a given size fraction. **(B)** Gene and transcript abundances. Values in the box plots correspond to the% of total diatom gene or transcript abundance, and are displayed in log_2_ scale.

Supplementary Figure 5 Biogeographical distribution of genes potentially involved in diatom carbon dioxide concentration mechanisms. Barplots are proportional to the gene abundance (% of the total diatom gene read abundance), while color indicates the enzyme: CA, carbonic anhydrase; CbbX, Rubisco activase; ME, malic enzyme; PEPC, phosphoenolpyruvate carboxylase, PEPCK, phosphoenolpyruvate carboxykinase. The Y axis shows the *Tara* Oceans stations and the ocean regions: MS, Mediterranean Sea; IO, Indian Ocean; SAO, South Atlantic Ocean; SO, Southern Ocean; SPO, South Pacific Ocean; NPO, North Pacific Ocean; NAO, North Atlantic Ocean.

Supplementary Figure 6 Biogeographical distribution of transcripts potentially involved in diatom carbon dioxide concentration mechanisms. Barplots are proportional to the transcript abundance (% of the total diatom transcript read abundance), while color indicates the enzyme. Abbreviations: CA, carbonic anhydrase; CbbX, Rubisco activase; ME, malic enzyme; PEPC, phosphoenolpyruvate carboxylase, PEPCK, phosphoenolpyruvate carboxykinase.

Supplementary Figure 7 Biogeographical distribution of genes coding for the different classes of diatom carbonic anhydrases. Barplots are proportional to the gene abundance (% of the total diatom gene read abundance), while color indicates the carbonic anhydrase class. The Y axis shows the *Tara* Oceans stations and the ocean regions. Abbreviations: MS, Mediterranean Sea; IO, Indian Ocean; SAO, South Atlantic Ocean; SO, Southern Ocean; SPO, South Pacific Ocean; NPO, North Pacific Ocean; NAO, North Atlantic Ocean.

Supplementary Figure 8 Biogeographical distribution of transcripts coding for the different classes of diatom carbonic anhydrases. Barplots are proportional to the transcript abundance (% of the total diatom transcript read abundance), while color indicates the carbonic anhydrase class. The Y axis shows the *Tara* Oceans stations and the ocean regions. Abbreviations: MS, Mediterranean Sea; IO, Indian Ocean; SAO, South Atlantic Ocean; SO, Southern Ocean; SPO, South Pacific Ocean; NPO, North Pacific Ocean; NAO, North Atlantic Ocean.

Supplementary Table 1 Sequences used in the current work. **(A)** List of Pfam models. **(B)** MATOU-v1 unigenes from diatoms coding for the five analyzed enzymes. **(C)** Sequences used for Supplementary Figure 1A. **(D)** Sequences and alignments used for Supplementary Figure 1B. **(E)** Sequences and alignments used for Supplementary Figure 1C. **(F)** Sequences used for Supplementary Figure 2.

Supplementary Table 2 Contextual data for the *Tara* Oceans samples used in the current work. Original source: https://doi.org/10.1594/PANGAEA.875582 and https://doi.pangaea.de/10.1594/PANGAEA.875567.

Supplementary Table 3 Abundance of genes and transcripts potentially involved in diatom carbon dioxide concentration mechanisms across the different *Tara* Oceans samples. Values correspond to the% of total diatom read abundance (in rpkm). carbonic anhydrase (CA), Rubisco activase (CbbX), malic enzyme (ME), phosphoenolpyruvate carboxylase (PEPC), and phosphoenolpyruvate carboxykinase (PEPCK).
