# Supplementary material for: Carbon Dioxide Concentration Mechanisms in Natural Populations of Marine Diatoms: Insights From Tara Oceans
Source: Front Plant Sci. 2021 Apr 30;12:657821. doi: 10.3389/fpls.2021.657821 (PMC8119650; doi:10.3389/fpls.2021.657821)
Supplement: Supplementary Figure 1 — Sequence analysis of CbbX and homologs. (A) Protein similarity network for the Pfam domain AAA (PF00004). Each node represents a given sequence and those sequences with similarity higher than a score cutoff are linked (score cut-off of 40 in blast alignment). The network was built with sequences retrieved from the literature and from reference genomes and transcriptomes. Nodes are colored according to their taxonomy. The cluster containing CbbX sequences and close homologs is circled. (B) Phylogeny of the Pfam domain AAA from the sequences belonging to the cluster highlighted in panel (A). The branch for CbbX is colored in yellow, whereas the remaining back branches are annotated as stage V sporulation protein K. (C) Phylogeny of the Pfam domain AAA from CbbX sequences, corresponding to the branch highlighted in panel (B). Color code varies according to the taxonomy. The sequence similarity network and the phylogenies were used as references for the selection of Tara Oceans unigenes encoding diatom CcbX. The list of sequences and the alignment are available in Supplementary Table 1. [file Data_Sheet_1.PDF]

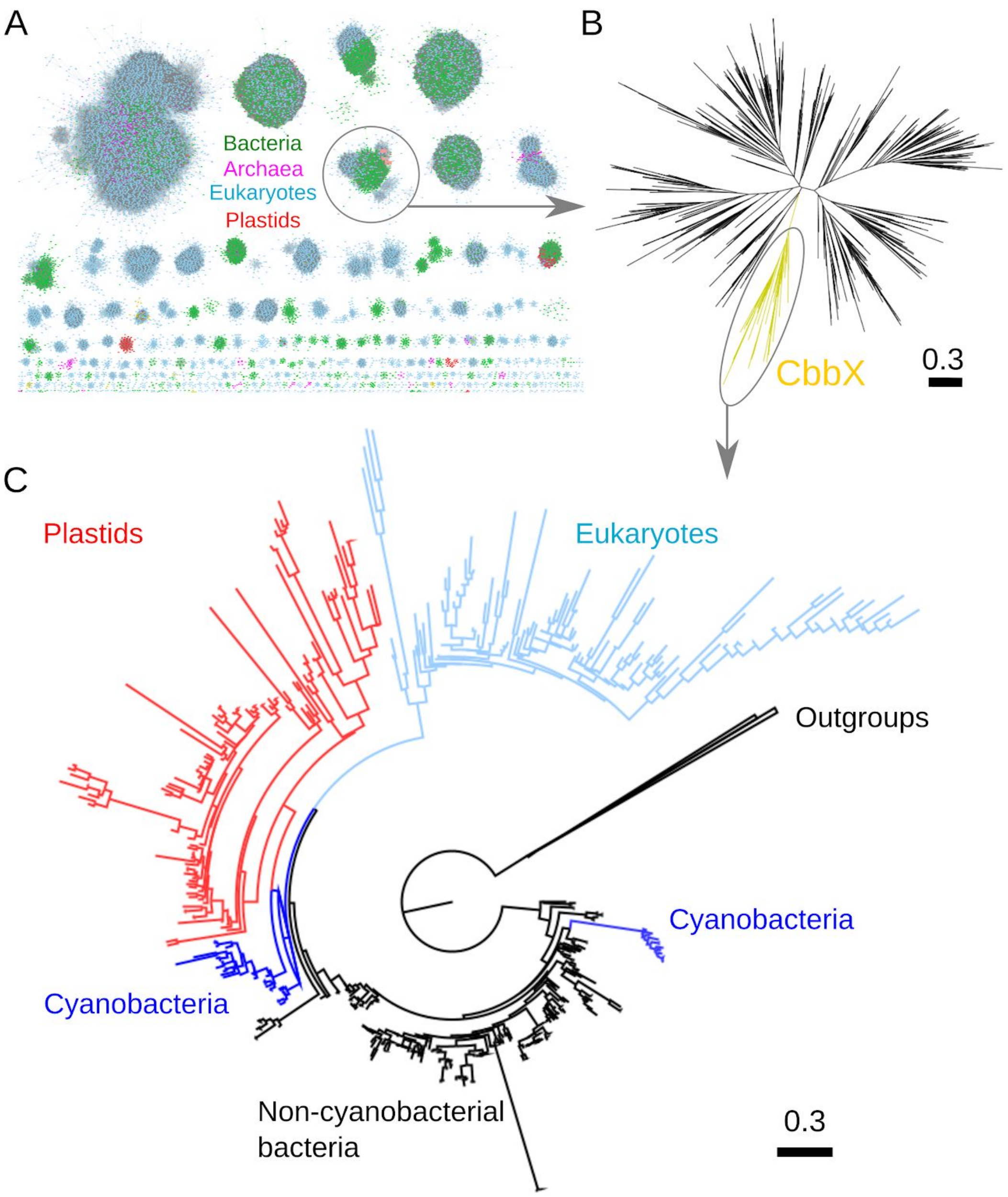

Supplementary Figure 1: Sequence analysis of CbbX and homologues. A) Protein similarity network for the Pfam domain AAA (PF00004). Each node represents a given sequence and those sequences with similarity higher than a score cutoff are linked (score cut-off of 40 in blast alignment). The network was built with sequences retrieved from the literature and from reference genomes and transcriptomes. Nodes are colored according to their taxonomy. The cluster containing CbbX sequences and close homologues is circled. B) Phylogeny of the Pfam domain AAA from the sequences belonging to the cluster highlighted in panel A. The branch for CbbX is coloured in yellow, whereas the remaining back branches are annotated as stage V sporulation protein K. C) Phylogeny of the Pfam domain AAA from CbbX sequences, corresponding to the branch highlighted in panel B. Color code varies according to the taxonomy. The sequence similarity network and the phylogenies were used as references for the selection of *Tara* Oceans unigenes encoding diatom CcbX. The list of sequences and the alignment are available in Supplementary Table S1.

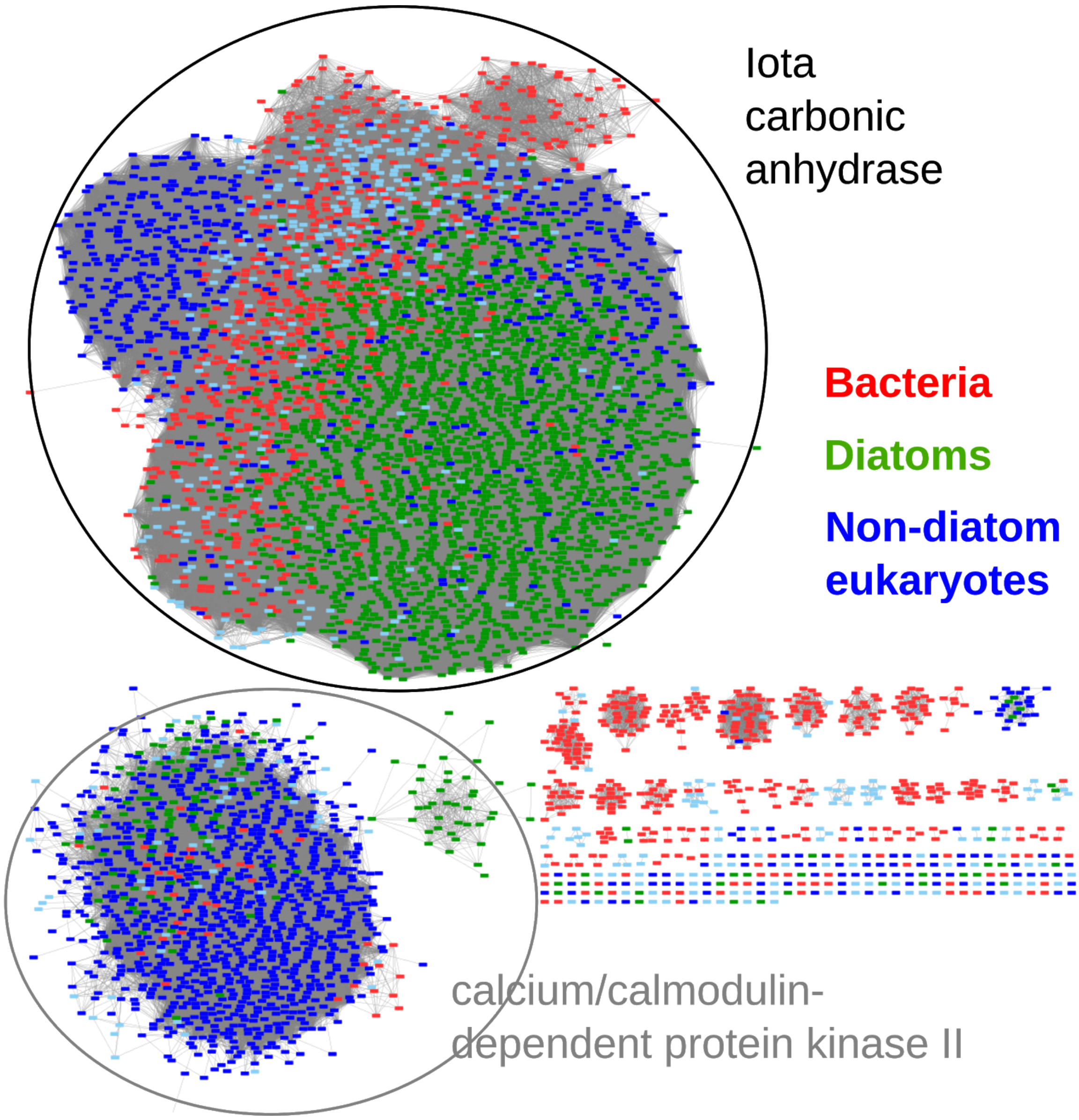

Supplementary Figure 2: Sequence analysis of iota carbonic anhydrase and homologues. Protein similarity network for the Pfam domain CaMKII_AD (PF08332). Each node represents a given sequence and those sequences with similarity higher than a score cutoff are linked (score cut-off of 18 in blast alignment). The network was built with sequences retrieved from the literature and from reference genomes and transcriptomes, as well as *Tara* Oceans unigenes. Nodes are coloured according to their taxonomy. The cluster containing the Iota carbonic anhydrase is circled, as well as the cluster containing calcium/calmodulin-dependent protein kinase II. The list of sequences is available in Supplementary Table S1.

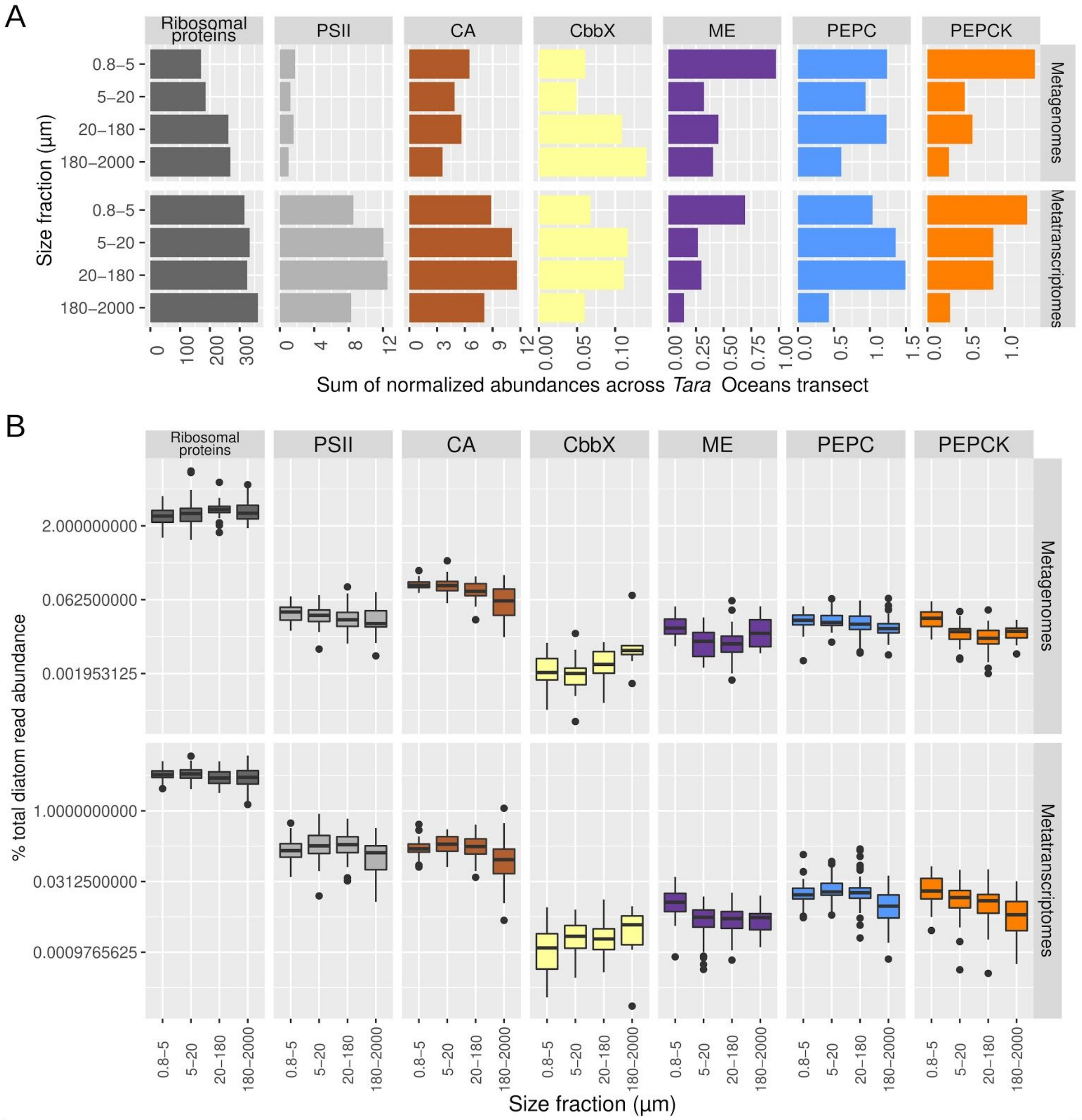

Supplementary Figure 3: Relative abundance of genes and transcripts potentially involved in diatom carbon dioxide concentration mechanisms in comparison to genes involved in other metabolisms. A) Sum of normalized abundances for all samples in a given size fraction. B) Gene and transcript abundance. Values in the box plots correspond to the % of total diatom gene or transcript abundance in the corresponding sample, and are displayed in $\log_2$ scale. In order to compare with other pathways, we also show the abundances for ribosomal proteins and for the nuclear-encoded subunits of photosystem II. Abbreviations: PSII, photosystem II; CA, carbonic anhydrase; CcbX, Rubisco activase; ME, malic enzyme; PEPC, phosphoenolpyruvate carboxylase; PEPCK, phosphoenolpyruvate carboxykinase.

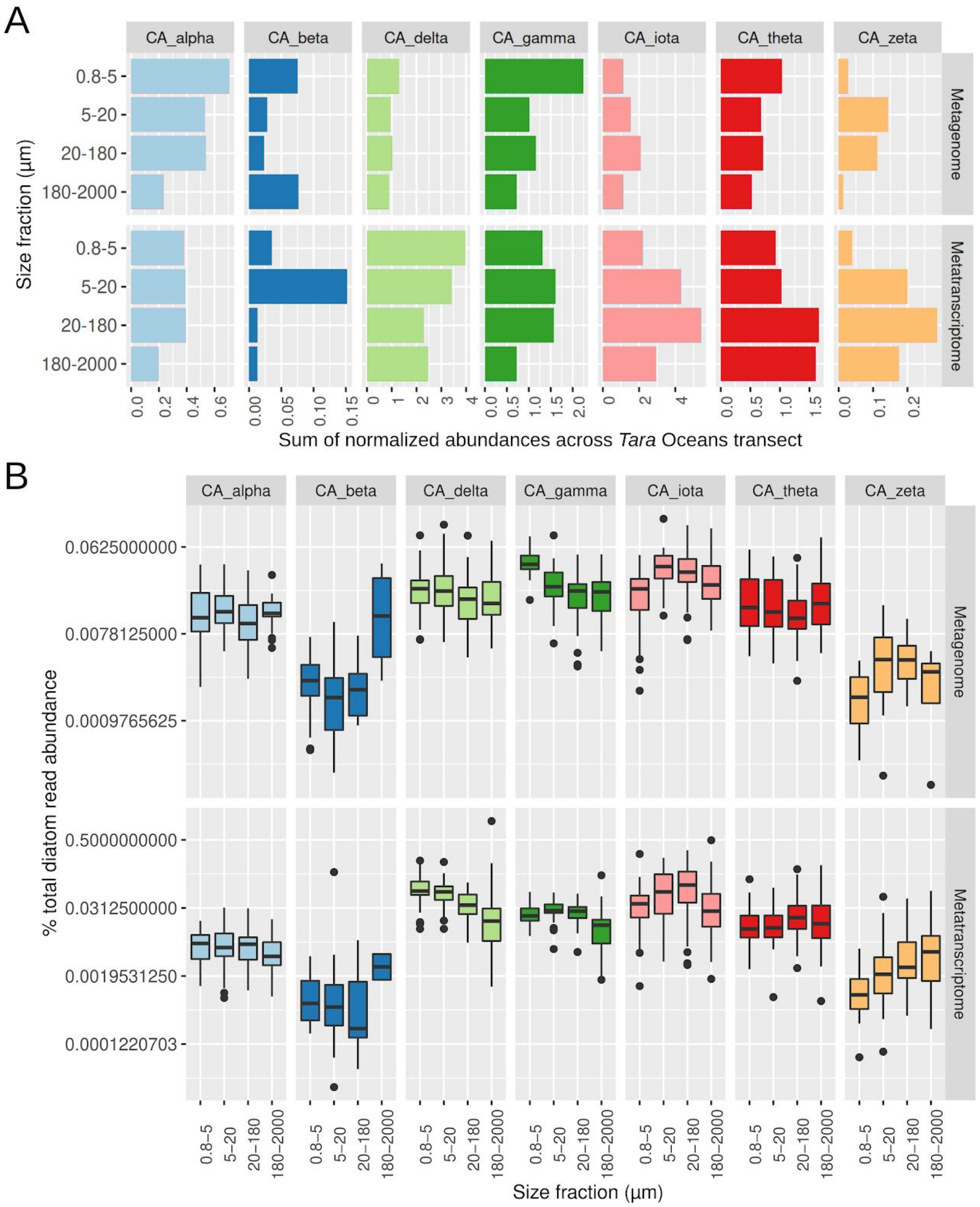

Supplementary Figure 4: Relative abundance of genes and transcripts coding for the different classes of diatom carbonic anhydrases across the size-fractionated seawater samples collected during the *Tara* Oceans transect. A) Sum of normalized abundances for all samples in a given size fraction. B) Gene and transcript abundance. Values in the box plots correspond to the % of total diatom gene or transcript abundance, and are displayed in logarithmic scale.

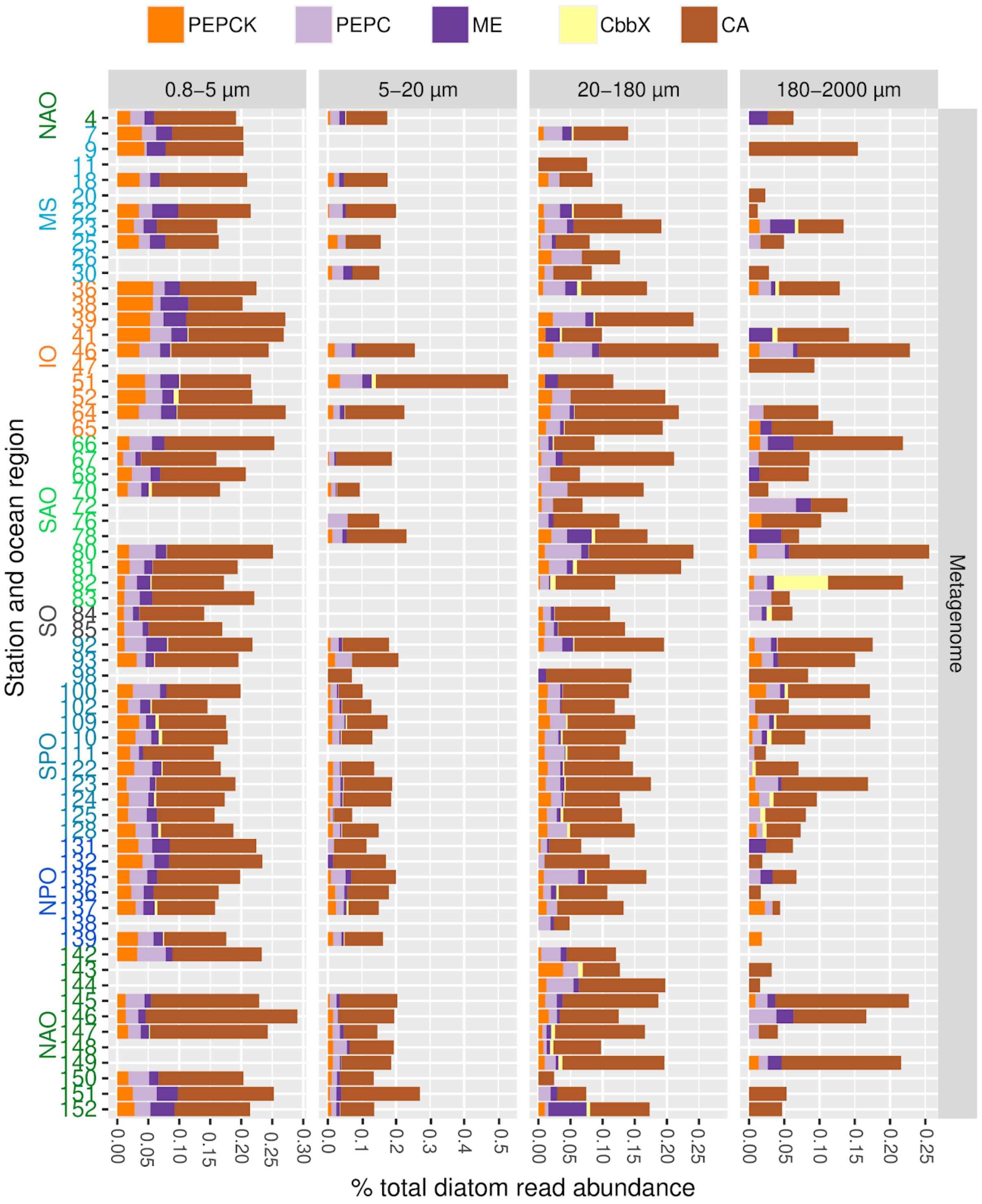

Supplementary Figure 5: Biogeographical distribution of genes potentially involved in diatom carbon dioxide concentration mechanisms. Barplots are proportional to the gene abundance (% of the total diatom gene read abundance), while colour indicates the enzyme: CA, carbonic anhydrase; CbbX, Rubisco activase; ME, malic enzyme; PEPC, phosphoenolpyruvate carboxylase, PEPCK, phosphoenolpyruvate carboxykinase. The Y axis shows the *Tara* Oceans stations and the ocean regions: MS, Mediterranean Sea; IO, Indian Ocean; SAO, South Atlantic Ocean; SO, Southern Ocean; SPO, South Pacific Ocean; NPO, North Pacific Ocean; NAO, North Atlantic Ocean.

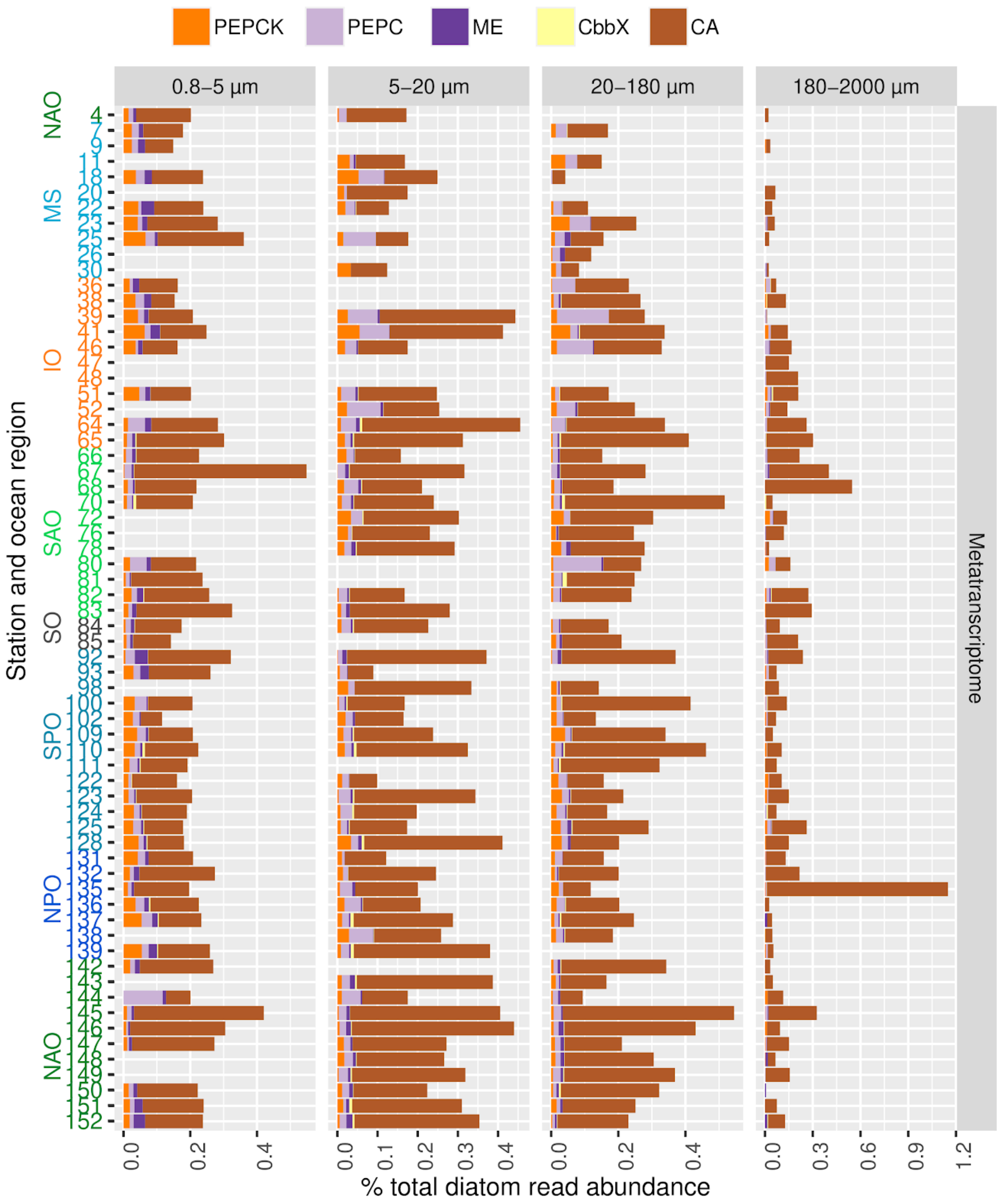

Supplementary Figure 6: Biogeographical distribution of transcripts potentially involved in diatom carbon dioxide concentration mechanisms. Barplots are proportional to the transcript abundance (% of the total diatom transcript read abundance), while colour indicates the enzyme. Abbreviations: CA, carbonic anhydrase; CbbX, Rubisco activase; ME, malic enzyme; PEPC, phosphoenolpyruvate carboxylase, PEPCK, phosphoenolpyruvate carboxykinase.

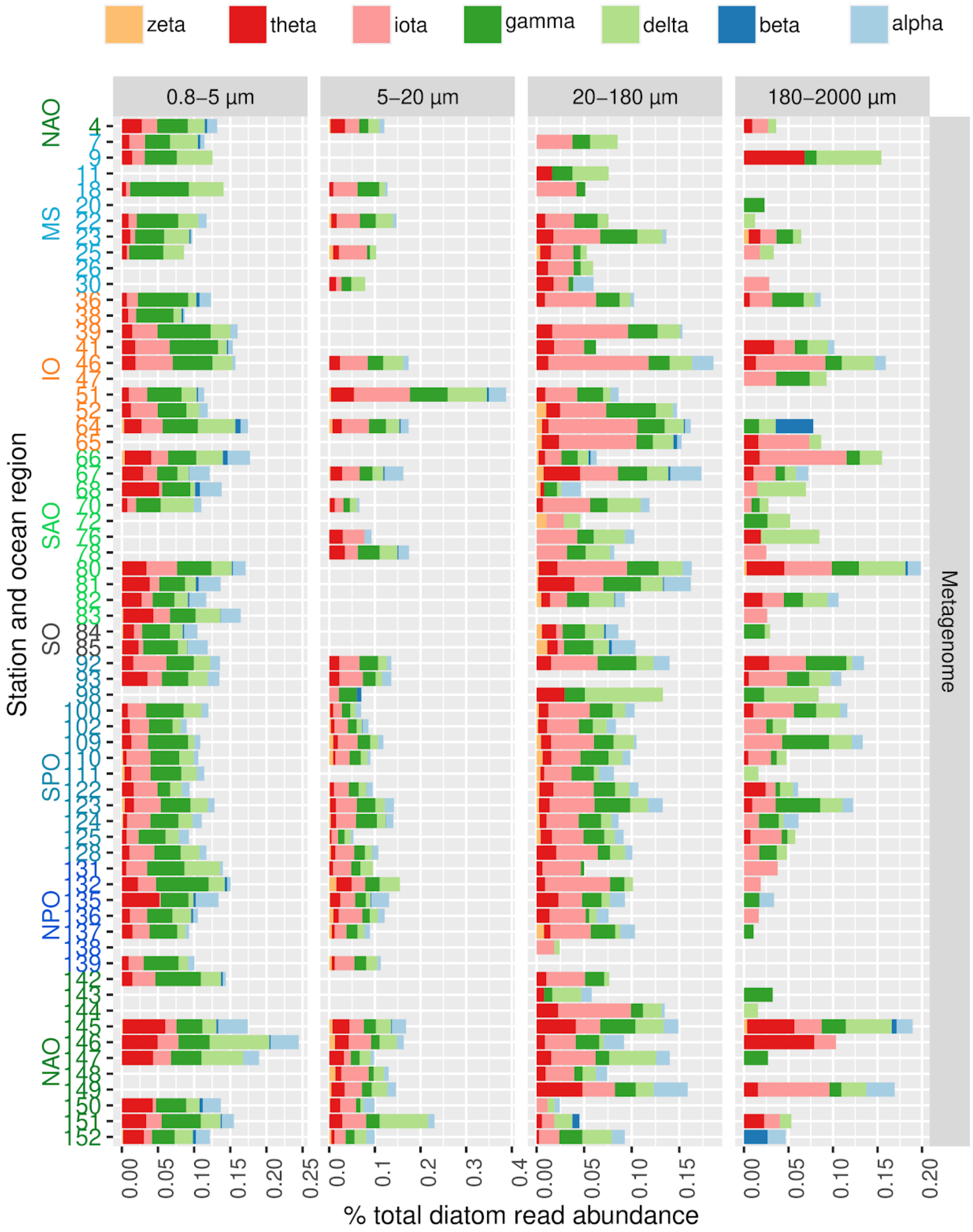

Supplementary Figure 7: Biogeographical distribution of genes coding for the different classes of diatom carbonic anhydrases. Barplots are proportional to the gene abundance (% of the total diatom gene read abundance), while colour indicates the carbonic anhydrase class. The Y axis shows the *Tara* Oceans stations and the ocean regions. Abbreviations: MS, Mediterranean Sea; IO, Indian Ocean; SAO, South Atlantic Ocean; SO, Southern Ocean; SPO, South Pacific Ocean; NPO, North Pacific Ocean; NAO, North Atlantic Ocean.

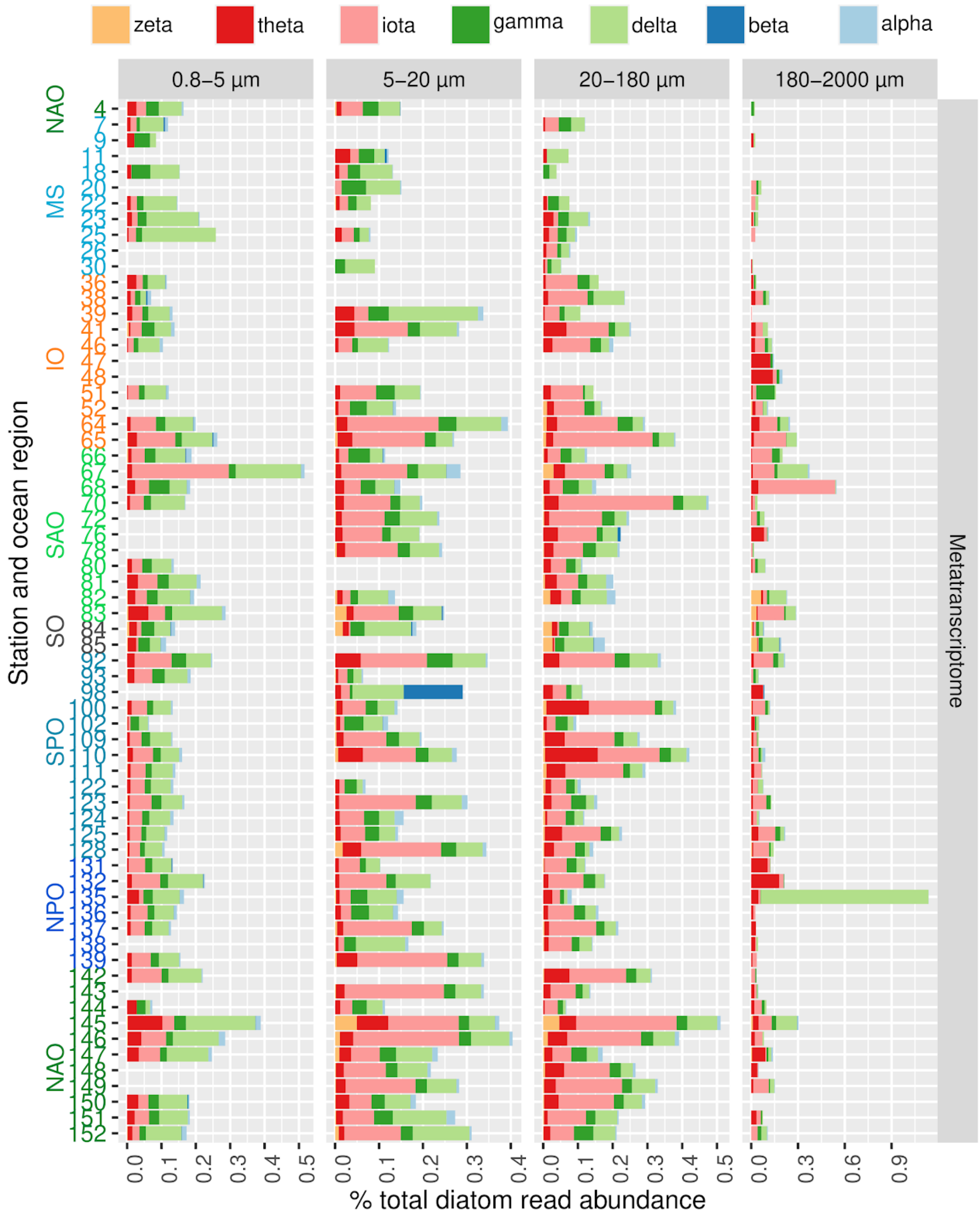

Supplementary Figure 8: Biogeographical distribution of transcripts coding for the different classes of diatom carbonic anhydrases. Barplots are proportional to the transcript abundance (% of the total diatom transcript read abundance), while colour indicates the carbonic anhydrase class. The Y axis shows the *Tara* Oceans stations and the ocean regions. Abbreviations: MS, Mediterranean Sea; IO, Indian Ocean; SAO, South Atlantic Ocean; SO, Southern Ocean; SPO, South Pacific Ocean; NPO, North Pacific Ocean; NAO, North Atlantic Ocean.
